# A Dynamic Interplay of Circulating Extracellular Vesicles and Galectin-1 Reprograms Viral Latency during HIV-1 Infection

**DOI:** 10.1128/mbio.00611-22

**Published:** 2022-08-09

**Authors:** Julia Rubione, Paula S. Pérez, Alejandro Czernikier, Gabriel A. Duette, Federico Pehuen Pereyra Gerber, Jimena Salido, Martina P. Fabiano, Yanina Ghiglione, Gabriela Turk, Natalia Laufer, Alejandro J. Cagnoni, Juan M. Pérez Sáez, Joaquín P. Merlo, Carla Pascuale, Juan C. Stupirski, Omar Sued, Manuel Varas-Godoy, Sharon R. Lewin, Karina V. Mariño, Gabriel A. Rabinovich, Matías Ostrowski

**Affiliations:** a Facultad de Medicina, Instituto de Investigaciones Biomédicas en Retrovirus y Sida (INBIRS), Universidad de Buenos Aires (UBA), Buenos Aires, Argentina; b Consejo Nacional de Investigaciones Científicas y Técnicas (CONICET), Buenos Aires, Argentina; c Laboratorio de Glicómica Funcional y Molecular, Instituto de Biología y Medicina Experimental (IBYME—CONICET), Buenos Aires, Argentina; d Laboratorio de Glicomedicina, Instituto de Biología y Medicina Experimental (IBYME—CONICET), Buenos Aires, Argentina; e Fundación Huésped, Buenos Aires, Argentina; f Cancer Cell Biology Lab, Centro de Biología Celular y Biomedicina (CEBICEM), Facultad de Medicina y Ciencia, Universidad San Sebastián, Santiago, Chile; g The Peter Doherty Institute for Infection and Immunity, The University of Melbourne and Royal Melbourne Hospital, Melbourne, Victoria, Australia; h Victorian Infectious Diseases Service, Royal Melbourne Hospital at the Peter Doherty Institute for Infection and Immunity, Melbourne, Victoria, Australia; i Department of Infectious Diseases, Alfred Health and Monash University, Melbourne, Victoria, Australia; j Facultad de Ciencias Exactas y Naturales, Universidad de Buenos Aires, Buenos Aires, Argentina; The Wistar Institute; Cornell University

**Keywords:** galectin-1, human immunodeficiency virus, extracellular vesicles, viral reservoir, chronic inflammation

## Abstract

Combined Antiretroviral therapy (cART) suppresses HIV replication but fails to eradicate the virus, which persists in a small pool of long-lived latently infected cells. Immune activation and residual inflammation during cART are considered to contribute to viral persistence. Galectins, a family of β-galactoside-binding proteins, play central roles in host-pathogen interactions and inflammatory responses. Depending on their structure, glycan binding specificities and/or formation of distinct multivalent signaling complexes, different members of this family can complement, synergize, or oppose the function of others. Here, we identify a regulatory circuit, mediated by galectin-1 (Gal-1)–glycan interactions, that promotes reversal of HIV-1 latency in infected T cells. We found elevated levels of circulating Gal-1 in plasma from HIV-1-infected individuals, which correlated both with inflammatory markers and the transcriptional activity of the reservoir, as determined by unspliced-RNA (US-RNA) copy number. Proinflammatory extracellular vesicles (EVs) isolated from the plasma of HIV-infected individuals induced Gal-1 secretion by macrophages. Extracellularly, Gal-1 interacted with latently infected resting primary CD4^+^ T cells and J-LAT cells in a glycan-dependent manner and reversed HIV latency via activation of the nuclear factor κB (NF-κB). Furthermore, CD4^+^ T cells isolated from HIV-infected individuals showed increased HIV-1 transcriptional activity when exposed to Gal-1. Thus, by modulating reservoir dynamics, EV-driven Gal-1 secretion by macrophages links inflammation with HIV-1 persistence in cART-treated individuals.

## INTRODUCTION

Human immunodeficiency virus (HIV) infection affects more than 38 million people worldwide, with 1.7 million new infections every year (1). HIV infection causes the progressive loss of CD4^+^ T cells, which, if left untreated, leads to AIDS. Interruption of the viral replication cycle by the administration of combined antiretroviral therapy (cART) stabilizes the CD4^+^ T cell count in most individuals, thus preventing immunodeficiency and reducing mortality. However, cART does not eradicate the virus, which persists in a pool of latently infected long-lived central memory and transitional memory CD4^+^ T cells that constitute the viral reservoir (1). This viral reservoir is responsible for viral rebound in the cases of cART interruption (2–5). Therefore, there is an urgent need to better understand the dynamics of reservoirs and to find strategies to directly target the latent virus in cART-treated individuals.

Alongside viral reservoirs, chronic immune activation constitutes a critical obstacle in the search for HIV cure and/or better therapeutic strategies (6). Inflammation underlies the pathogenesis of a series of non-AIDS-related morbidities, including neoplastic, cardiovascular, neurologic, kidney, and bone diseases (7–12). Indeed, inflammatory markers, mostly associated with macrophage activity, such as interleukin 6 (IL-6), IP-10, soluble CD163 (sCD163), and sCD14, can predict the development of these pathologies in HIV-1-infected individuals and strongly correlate with disease severity and mortality (13–15). Several mechanisms have been proposed to explain the development of chronic inflammation in cART-treated individuals, including a breach of the mucosal barrier (16, 17), and loss of regulatory T cells (Tregs) and fibrosis of primary or secondary lymphoid organs (18), as well as responses to other pathogens, such as cytomegalovirus (19). Importantly, heightened inflammation is associated with the size of the viral reservoir (1, 20–22). In this regard, it has been suggested that inflammation enhances viral production or increases the number of activated CD4^+^ T cells, thus expanding the number of susceptible target cells (23). Hence, a better understanding of the inflammatory mediators underlying viral rebound may contribute to designing novel therapeutic strategies aimed at reducing the size of the viral reservoirs (23–25; https://clinicaltrials.gov/ct2/show/NCT02440789).

Extracellular vesicles (EVs) are small membrane particles (with diameters ranging from tens to hundreds of micrometers) limited by a lipid bilayer and released by almost all cell types into their extracellular environment (26). By transferring proteins, lipids, and nucleic acids into target cells, EVs mediate intercellular communication (27) and have been implicated in the pathogenesis of several clinical manifestations, such as cancer and a variety of infectious diseases, including HIV infection (28–31). We have recently shown that circulating EVs isolated from the plasma of HIV-infected individuals can activate macrophages and promote the secretion of proinflammatory cytokines (32), thus contributing to HIV-related chronic inflammation. Nevertheless, how EV-mediated inflammation could modulate HIV reservoir dynamics is still an unanswered question.

Galectins are a family of soluble lectins, capable of recognizing *N*-acetyllactosamine (Galβ1-4-GlcNAc; LacNAc) residues on a broad repertoire of glycosylated cell surface receptors (33, 34). When analyzing galectins from a functional perspective, it can be observed that individual members of this family may either complement, synergize, or oppose the function of others by virtue of structural differences, variations in glycan binding specificities, and/or formation of distinct multivalent signaling complexes (35, 36). Whereas prototype galectins (including galectin-1 [Gal-1]) function as noncovalent homodimers with one carbohydrate recognition domain (CRD) in each monomer, tandem-repeat galectins (including galectin-9 [Gal-9]) have two CRDs joined by a flexible linker peptide, and the chimera-type galectin-3 (Gal-3) is composed of a nonlectin domain linked to a CRD. Although they all share recognition of LacNAc derivatives, Gal-1 recognizes terminal LacNAc structures both in N-glycans and in core 2 O-glycans, Gal-9 prefers LacNAc repetitive sequences and glycosphingolipids, and Gal-3 can recognize internal LacNAc structures in sialylated and nonsialylated glycans (37). This evidence, indicating critical differences in structure and glycan binding preferences, suggests autonomous functions of individual galectins in the establishment of regulatory circuits and the control of pathophysiologic processes. In fact, emerging evidence suggests that targeting the galectin-glycan axis may contribute to control of infections caused by a range of sexually transmitted pathogens (38).

Within the immune system, galectins can control innate and adaptive immune cell programs (39), either as proresolving (40–42) or as proactivating (43, 44) factors, by differentially regulating the fate and function of lymphoid and myeloid cells. Particularly, in the context of HIV infection, Gal-1 and Gal-3 can influence viral attachment to CD4^+^ T cells and replication (45, 46), whereas Gal-9 has been implicated in the control of HIV latency (47). However, the role of Gal-1 and its glycosylated ligands in reprogramming viral reservoir dynamics and the underlying inflammatory response has not yet been explored.

Notably, plasma Gal-1 levels are elevated in a broad range of inflammatory settings, including sepsis (44), rheumatoid arthritis (48), and different cancer types (49), highlighting the potential role of this lectin as a prognostic biomarker. Since Gal-1 profoundly influences CD4^+^ T cell functionality (50) and macrophages are an important source of this lectin (51), we investigated the interplay between macrophage activation and Gal-1 secretion during HIV-1 infection and its impact on latently infected CD4^+^ T cells. Here, we provide *in vitro* and *in vivo* evidence showing that in response to EVs circulating in the plasma of HIV-1-infected individuals, human macrophages secrete Gal-1, which promotes glycan-dependent HIV-1 latency reversal. Our results highlight the interconnected role of EVs and Gal-1 in the control of reservoir dynamics, suggesting therapeutic possibilities for manipulating Gal-1 expression to modulate the viral reservoir in the HIV-1-infected population.

## RESULTS

### Increased levels of circulating Gal-1 are associated with inflammation and reservoir transcriptional activity in HIV-1-infected individuals.

**FIG 1:**
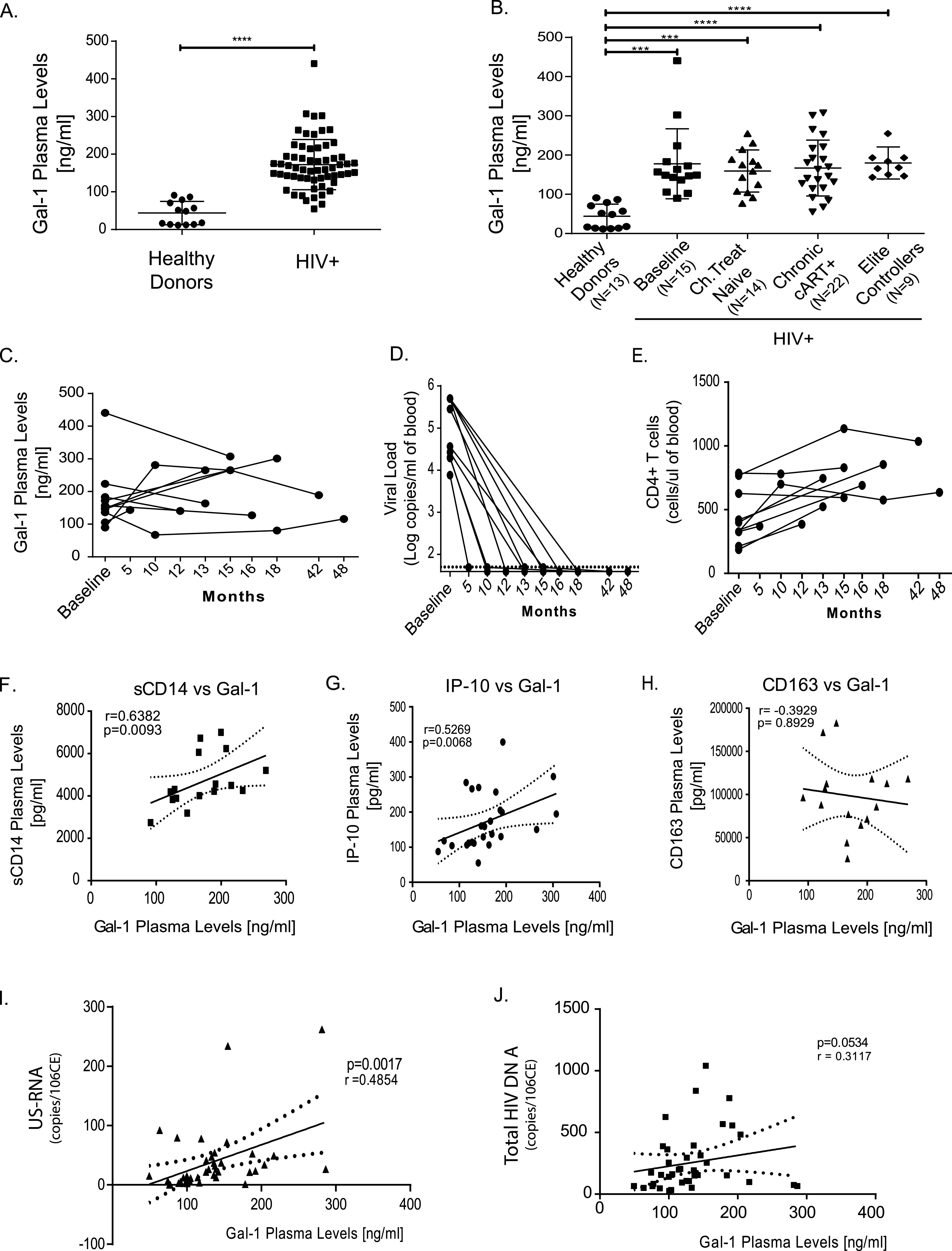
Gal-1 is elevated in the plasma of HIV-1-infected individuals. (A) Concentrations of Gal-1 in the plasma of 13 HIV-1-negative and 51 HIV-1-positive individuals were determined by ELISA. (B) HIV-1-infected individuals were classified according to the course of infection and treatment status. (C) Changes in plasma levels of Gal-1 over time in 10 cART-treated individuals. The first blood sample was obtained at the time of diagnosis and before initiation of cART treatment (baseline), and subsequent samples were obtained at the indicated months after initiation of cART treatment. Serum Gal-1 levels were determined by ELISA. (D and E) Virological and immunological status associated with cART treatment were evaluated in subjects analyzed for panel C by determination of viral load (D) and CD4^+^ T cell counts (E) at different times after treatment initiation. (F to H) Correlation analysis between Gal-1 levels present in serum samples from HIV-infected individuals under cART and inflammatory markers, including sCD14 (F), IP-10 (G), and sCD163 (H). (I and J) Correlation between Gal-1 levels present in serum samples from HIV-infected individuals under cART and reservoir size, as determined by average copy number of unspliced HIV RNA (I) and average HIV DNA copies (J). *P* values were calculated using the Mann-Whitney and Kruskal-Wallis tests (A and B, respectively). ***, *P* < 0.001; ****, *P* < 0.001. (F to J) *r* value and *P* value correspond to Spearman’s test.

To investigate the role of Gal-1 in HIV-1 pathogenesis, we first studied serum concentrations of this lectin during HIV infection. By evaluating 69 samples from 13 healthy HIV-seronegative donors and 51 HIV-infected individuals (see Table S1 in the supplemental material), we detected considerably larger amounts of circulating Gal-1 in HIV-1-infected than in uninfected individuals (*P* < 0.0001 [Fig. 1A]). To better understand the spatiotemporal and context-dependent regulation of this glycan-binding protein, we classified our study group according to the course of the disease (early versus chronic infection) and to treatment status (untreated or on cART). In addition, we analyzed a group of elite controllers, defined as individuals with undetectable viral load and preserved CD4^+^ T cell counts (median = 869.5 cells/mm^3^; interquartile range from 25 to 75% [IQ25-75%] = 760.75 to 942) in the absence of cART. Thus, four groups were constituted: (i) early infection, baseline (treatment naive); (ii) chronic infection, treatment naive; (iii) chronic infection, on cART; and (iv) elite controllers. Remarkably, we observed that despite highly significant differences in viral load and CD4^+^ T cell numbers (Table S1), serum concentrations of Gal-1 were similar in all four groups analyzed (Fig. 1B), suggesting that the HIV-1-associated increase of Gal-1 is independent of viral load and immunological status. This was further confirmed by a longitudinal analysis of serum Gal-1 levels over time (between 12 and 48 months) in 10 individuals. Samples were obtained before initiation of cART (baseline) and at different periods following initiation of treatment. We observed that Gal-1 serum concentrations did not change significantly over time within each individual (Fig. 1C), even though cART treatment was effective, as evidenced by a dramatic decrease in viral load (Fig. 1D) and a sustained increase in CD4^+^ T cell count (Fig. 1E). Thus, increased concentrations of Gal-1 in sera from HIV-1-infected individuals are independent of changes in viral load, CD4^+^ T cell count, and treatment status.

10.1128/mbio.00611-22.8 TABLE S1 Clinical features of patients included in this study. Patient ID, Gal-1 plasma concentration, viral load (copies per milliliter), CD4 T cell count (cells per milliliter), gender, age, and presentation of AIDS-related diseases are included. (a) HD, healthy donor; B, baseline; ChTN, chronic treatment naive; ChcART, chronic under cART; EC, elite controller. (b) Determinations by ELISA. (c) Diagnosed prior to HIV infection. F, female; M, male; NA, not available. Download Table S1, PDF file, 0.1 MB. Copyright © 2022 Rubione et al. 2022 Rubione et al. https://creativecommons.org/licenses/by/4.0/ This content is distributed under the terms of the Creative Commons Attribution 4.0 International license.

To expand our results further beyond this local cohort, we conducted a meta-analysis on publicly available transcriptomics data from HIV-infected patients (52). First, we narrowed all available studies down to 14 targeted studies including 15 data sets (Table S2) and analyzed *LGALS1* (encoding Gal-1) expression. In 10 out of 15 data sets included, *LGALS1* mRNA was increased (fold change >1) in infected individuals, even in those undertaking cART (Fig. S1). Supporting our findings (Fig. 1C to E), no significant changes were observed in *LGALS1* mRNA expression in peripheral blood mononuclear cells (PBMCs) from HIV-infected individuals at baseline and after 1 year of initiation of cART (Bl versus cART condition in study 11 [Fig. S1]). Taken together, our results and meta-analysis of published data demonstrate that HIV infection itself is associated with increased transcription and secretion of Gal-1. Interestingly, as revealed by our meta-analysis, *LGALS1* mRNA was particularly upregulated in myeloid cells isolated from subjects under cART, suggesting that these cells could be a major source of this lectin.

10.1128/mbio.00611-22.1 FIG S1 Meta-analysis of publicly available transcriptional data sets demonstrates associations between HIV infection and *LGALS1* mRNA expression. Heat map showing log fold change of *LGALS1* mRNA expression of HIV^+^ samples over HIV^−^ samples for different tissues over 15 data sets. Fold changes are depicted inside the boxes. cART, combined antiretroviral therapy; Bl, Baseline. Download FIG S1, EPS file, 2.3 MB. Copyright © 2022 Rubione et al. 2022 Rubione et al. https://creativecommons.org/licenses/by/4.0/ This content is distributed under the terms of the Creative Commons Attribution 4.0 International license.

10.1128/mbio.00611-22.9 TABLE S2 Studies included in *LGALS1* mRNA meta-analysis. Sample set, accession number, and sample count per study are shown. Download Table S2, PDF file, 0.1 MB. Copyright © 2022 Rubione et al. 2022 Rubione et al. https://creativecommons.org/licenses/by/4.0/ This content is distributed under the terms of the Creative Commons Attribution 4.0 International license.

We next explored the association between serum Gal-1 concentrations and three inflammatory markers previously shown to be increased in cART-treated individuals (53–57). We observed a positive correlation between serum levels of Gal-1 and sCD14 (Fig. 1F) and IP-10 (Fig. 1G) but did not find any correlation with sCD163 (Fig. 1H).

Finally, we investigated the association between serum Gal-1 concentrations and the reservoir size in circulating CD4^+^ T cells, as estimated by the copy numbers of unspliced RNA (US-RNA) or total viral DNA (57, 58) (Table S3). Interestingly, Gal-1 serum levels positively correlated with US-RNA in CD4^+^ T cells (Fig. 1I). In contrast, we did not observe any correlation between Gal-1 concentrations and total DNA copy numbers, although a trend toward an association between Gal-1 serum levels and DNA copies was observed (Fig. 1J). Given that total DNA reflects the size of the proviral reservoir, whereas US-RNA levels mirror HIV-1 transcriptional activity during effective treatment, these results suggest that increased circulating Gal-1 could promote HIV-1 transcription *in vivo*.

10.1128/mbio.00611-22.10 TABLE S3 Clinical and virological data of patients. Patient ID, duration of treatment (days), total HIV-1 DNA (average copy number/10^6^ cell equivalents) and unspliced RNA (average copy number/10^6^ cell equivalents), viral load (copies per milliliter), CD4 T cell count (cells per milliliter), and Gal-1 plasma concentration (nanograms per milliliter) are included. Download Table S3, PDF file, 0.02 MB. Copyright © 2022 Rubione et al. 2022 Rubione et al. https://creativecommons.org/licenses/by/4.0/ This content is distributed under the terms of the Creative Commons Attribution 4.0 International license.

Thus, plasma from HIV-1-infected individuals contains increased levels of circulating Gal-1, which are independent of viral load or CD4^+^ T cell counts but are associated with inflammation and with HIV-1 transcriptional activity.

### Extracellular vesicles in plasma from HIV-1-infected individuals induce Gal-1 secretion by macrophages.

**FIG 2:**
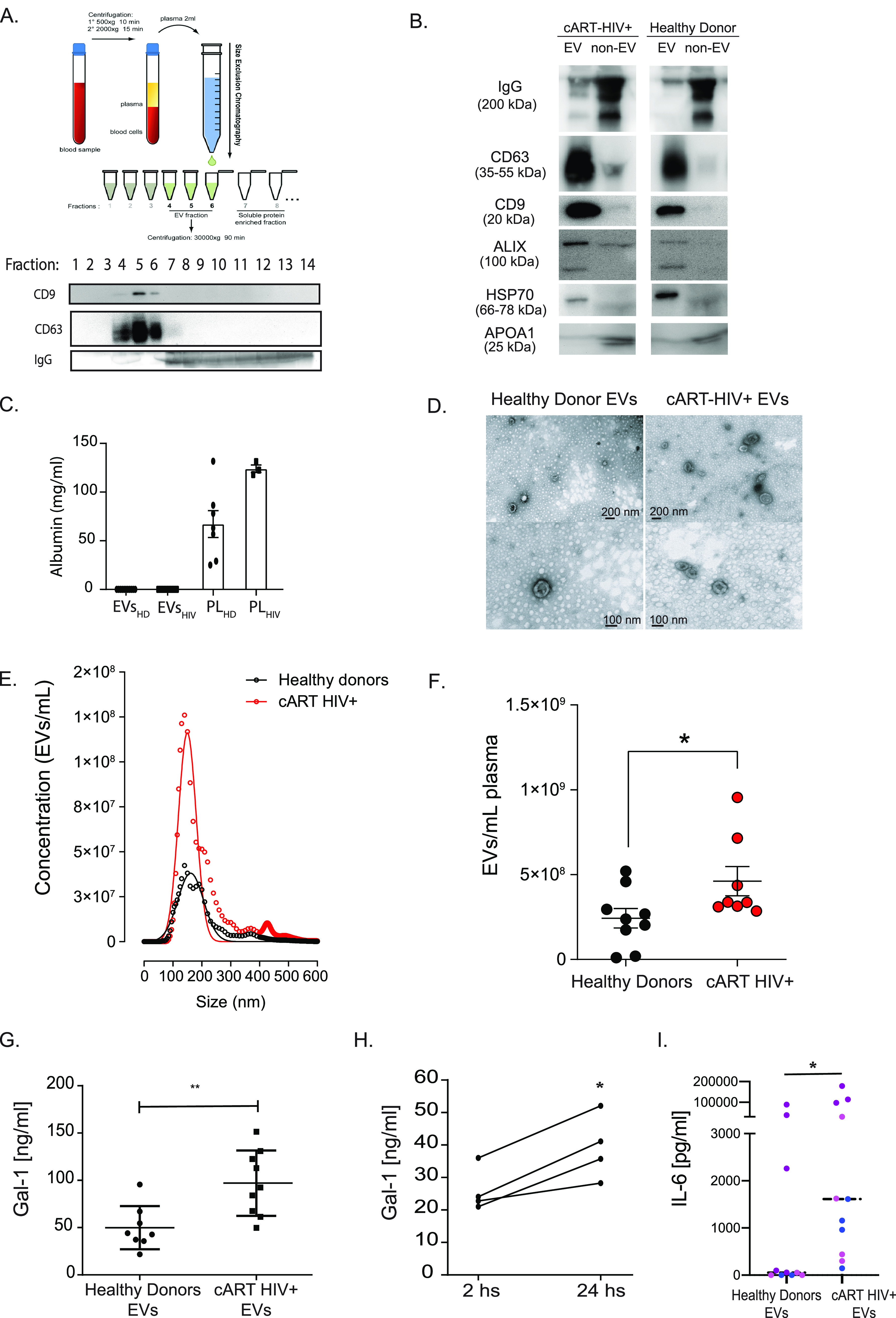
Extracellular vesicles (EVs) circulating in the plasma of HIV-1-infected individuals trigger Gal-1 secretion by macrophages. (A) EVs were isolated by SEC followed by centrifugation from plasma of either healthy controls or HIV-1-infected individuals. The presence and purity of EVs in each fraction collected were analyzed by immunoblotting. Fractions 4 to 6 (enriched in EV markers) were pooled and centrifuged at 30,000 × *g*, and the pellet containing the EVs was resuspended in PBS. Illustration previously published in reference (32). (B) Characterization of plasma EVs by immunoblot analysis. Equal amounts of EV fractions (4 to 6) and non-EV fractions (7 to 9) from cART-treated HIV^+^ or healthy donor plasma were analyzed for CD63, CD9, ALIX, and HSP70 (inclusion markers) or IgG and APOA1 (exclusion markers). (C) Albumin was determined by immunoturbidimetry on plasma EV preparations from cART-treated HIV^+^ or healthy donor plasma and on plasma samples (PL) as positive controls. (D) Transmission electron microscopy (TEM) micrographs showing EVs purified by SEC combined with centrifugation at 30,000 × *g* from plasma of cART-treated HIV^+^ and healthy donors. Scale bars are shown for all micrographs. (E) Size distribution of plasma EVs from healthy and HIV^+^ cART-treated donors obtained by NTA, showing mean concentration (*n* = 9) for each EV size (circles) and the nonlinear regression fit (line). (F) Determination by NTA of the concentration (means ± SEM) of EVs isolated by SEC followed by centrifugation in plasma from healthy (black, *n* = 9) or cART-treated HIV^+^ (red, *n* = 8) donors. (G) Purified EVs from the plasma of either uninfected or HIV-1-infected individuals were added to MDM cultures (2.5 × 10^5^/well). The presence of Gal-1 in cell culture supernatant was determined by ELISA at day 1 after addition of EVs. (H) Kinetics of Gal-1 production by MDMs stimulated with EVs isolated from plasma of HIV-1-infected individuals. (I) IL-6 production and release by MDMs stimulated for 24 h with EVs isolated from the plasma of HIV-1-infected individuals was evaluated in cell culture supernatants by ELISA. Mean values segregated by MDM donors are shown. In total, samples from 8 healthy EV donor and 11 HIV^+^ cART-treated EV donors were analyzed. *P* values were calculated using unpaired (G to I) or paired (H) Student’s *t* test. *, *P* < 0.05; **, *P* < 0.01.

Since EVs isolated from plasma of HIV-1-infected individuals stimulate monocyte-derived macrophages (MDMs) to release proinflammatory cytokines (32), and our results showed an association between elevated Gal-1 levels and inflammatory parameters in sera during HIV infection (Fig. 1), we next analyzed whether EVs isolated from plasma of HIV-1-infected individuals (EV_HIV_) could trigger Gal-1 production by macrophages. EV_HIV_ or EVs from healthy donors (EV_HD_) were isolated by size exclusion chromatography (SEC) as previously described (32). This fractionation method is based on the size of plasma components: large components such as EVs do not reach the packed matrix pores and are eluted first, whereas soluble proteins are trapped inside the matrix pores and elute later. The purity of EVs eluted in fractions 4 to 6 was assessed by immunoblot analysis; the results showed that the EV-containing fractions were positive for tetraspanins CD63 and CD9 and did not include soluble IgG, which was eluted in later fractions (Fig. 2A). Further characterization revealed that all EV preparations, from HIV^+^ and healthy individuals, were enriched in canonical EV markers, including tetraspanins CD63 and CD9 (membrane-associated proteins) and cytosolic proteins, including ALIX and HSP70, compared to the non-EV fractions (Fig. 2B). In contrast, plasma soluble IgG, readily detectable in the non-EV fractions, was mostly excluded from the EV preparations. In addition, very low concentrations of albumin (less than 0.1 mg/mL) were detected in EV fractions (Fig. 2C), indicating that the EV isolation method was highly effective in removing plasma soluble proteins. Finally, APOA1, a high-density lipoprotein (HDL) marker, was mostly present in the non-EV fraction (Fig. 2B), indicating very low levels of contaminant lipoproteins in the EV fractions. Since EVs and HIV virion particles could be coisolated by SEC, in this study, we used samples from HIV-infected individuals on cART with undetectable viral load. However, to verify that our EV preparations were free of viral particles that could complicate the interpretation of the results, the presence of HIV RNA was investigated on EV preparations by quantitative PCR (qPCR). As expected, donors with undetectable HIV-1 in plasma also showed undetectable HIV-1 RNA on isolated EVs (Table 1). In addition, transmission electron microscopy (TEM) visualization showed the characteristic double-membrane cup-shaped structures in both healthy and HIV^+^ EV samples (Fig. 2D). To further characterize our EV preparations, we analyzed EV size distribution and concentration by different particle analysis platforms, including nano-tracking analysis (NTA) (Fig. 2E and F), dynamic light scattering (DLS) (Fig. S2A), and microfluidics resistive pulse sensing (MRPS) (Fig. S2B and C). EV_HD_ and EV_HIV_ were comparable in size as determined by all methodologies applied. NTA showed mean diameters of 199.0 ± 6.6 nm and 196.9 ± 3.5 nm and mode diameters of 146.5 ± 7.0 nm and 142.1 ± 3.5 nm for EV_HD_ and EV_HIV_, respectively, which were not significantly different. The EV concentrations measured by NTA on isolated EVs and calculated in relation to the original plasma sample were 2.4 ± 0.6 × 10^8^ and 4.6 ± 0.9 × 10^8^ EVs/mL for EV_HD_ and EV_HIV_, respectively, showing a slight increase in circulating EVs in HIV^+^ individuals (1.9-fold). Taken together, these data indicate that plasma EVs from healthy and HIV-positive individuals have similar protein compositions, show comparable diameters, and are present at similar frequencies in the circulation.

**TABLE 1:** HIV-1 viral load in EVs^*a*^

Patient	Plasma viral load (copies/mL)	EV viral load (copies/mL)
1	Undetected	<20
2	<20	<20
3	Undetected	Undetected
4	Undetected	Undetected
5	Undetected	Undetected
Positive control	123,000	42,600

a HIV RNA was investigated on EV preparations by standard methods used for viral load determination in patients’ plasma samples.

10.1128/mbio.00611-22.2 FIG S2 Characterization of plasma EVs. (A) Size distribution of plasma EVs from healthy and HIV^+^ cART donors obtained by DLS. Three representative measure replicates are shown for each sample. D, mean hydrodynamic diameter ± SD; PDI, polydispersity index ± SD. (B) Particle size distribution of plasma EVs (above 80 nm) was analyzed by MRPS. The histogram shows EV concentration particles for each individual size in nanometers normalized against the maximum frequency for plasma EVs from healthy (gray lines, *n* = 3) or cART-treated HIV^+^ (red lines, *n* = 3) donors isolated by SEC followed by centrifugation. (C) Concentration of plasma EVs (mean ± SEM) above 80 nm from healthy (black, *n* = 3) or cART-treated HIV^+^ (red, *n* = 3) donors isolated by SEC followed by centrifugation and analyzed by MRPS. ns, not significant. Download FIG S2, EPS file, 2.4 MB. Copyright © 2022 Rubione et al. 2022 Rubione et al. https://creativecommons.org/licenses/by/4.0/ This content is distributed under the terms of the Creative Commons Attribution 4.0 International license.

We next analyzed the functionality of EVs isolated from HIV-infected individuals and from healthy donors by stimulating MDMs and monitoring the production of Gal-1. We observed that MDMs incubated with EV_HIV_ secreted larger amounts of Gal-1 than did MDMs incubated with EV_HD_ (Fig. 2G). Moreover, the amounts of secreted Gal-1 increased with longer incubation periods (Fig. 2H), suggesting that Gal-1 is actively produced by MDMs following stimulation with EVs. As expected (32), EV_HIV_ also triggered IL-6 production by MDMs (Fig. 2I). Thus, EVs circulating in the plasma of HIV-1-infected individuals stimulate the release of Gal-1 along with IL-6 from macrophages, suggesting a regulatory role for this glycan-binding protein during HIV-1 infection and persistent immune activation.

### Gal-1 secreted by EV-stimulated macrophages reverses HIV-1 latency.

**FIG 3:**
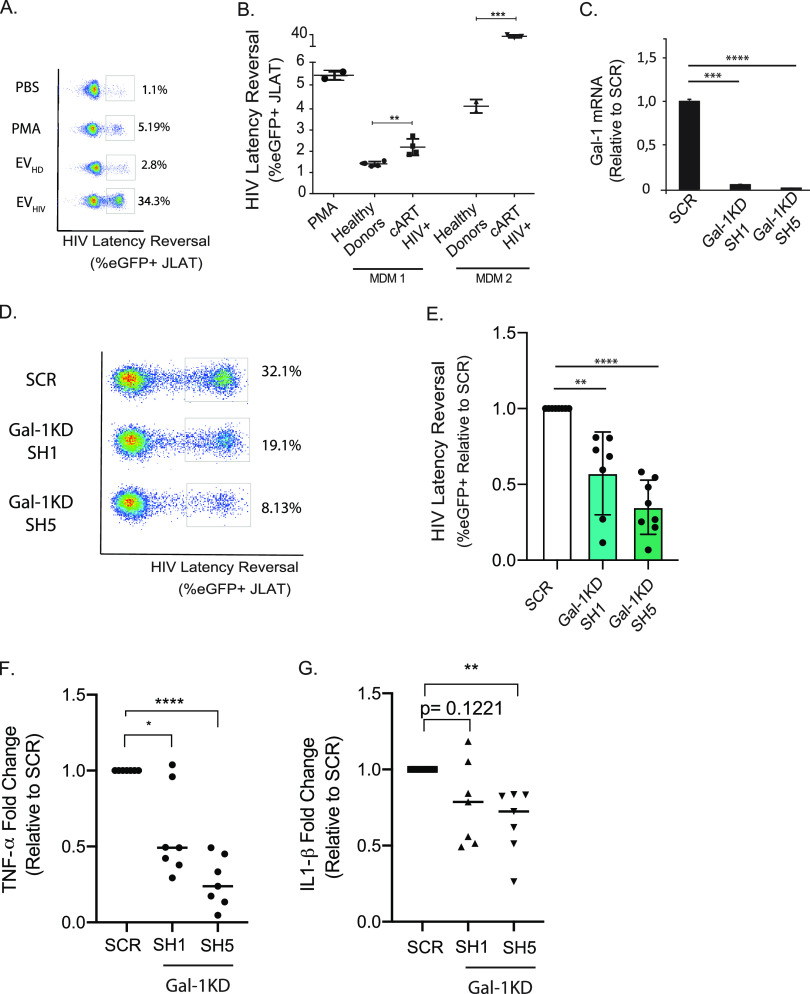
Gal-1 produced by EV-stimulated macrophages activate HIV-1 latency. (A and B) MDMs were stimulated with EVs isolated from the plasma of either uninfected or HIV-1-infected individuals for 24 h. Conditioned medium was collected, centrifuged to remove cells and cell debris, and added to cultures of J-LAT cells (3 × 10^5^; clone 10.6). Latency reversal was determined 24 h later by determining the percentage of eGFP-positive J-LAT cells by FACS. (A) Representative dot plots depicting eGFP expression in cells treated with MDM supernatants after EV_HD/HIV_ stimulation are shown together with negative (PBS) and positive (PMA) controls (A). Results from two independent experiments in which 8 and 5 EV_HIV_ donors were analyzed using two independent MDM cultures, respectively (B), are shown. (C) Expression of Gal-1 in MDMs was silenced by shRNA. *LGALS1* mRNA levels were evaluated by qPCR. Pooled data from two donors are shown. (D and E) Gal-1-silenced MDMs were stimulated with EV_HIV_ and conditioned medium was used to stimulate J-LAT cells (3 × 10^5^ cells, clone 10.6). Representative dot plots (D) and pooled data (normalized to scrambled) from three independent experiments including 8 EV donors and MDMs from three different donors (E) are shown. (F and G) TNF-α and IL-1β concentrations were determined in conditioned medium from panel E by a cytokine bead array. *P* values were calculated using unpaired Student’s *t* test (B) or one-way ANOVA (C, E, F, and G). *, *P* < 0.05; **, *P* < 0.01; ***, *P* < 0.001; ****, *P* < 0.0001.

Given the association of Gal-1 with the transcriptional activity of the HIV reservoir (Fig. 1I), we hypothesized that Gal-1 released by EV-stimulated macrophages could reprogram viral latency. To evaluate this hypothesis, conditioned medium from MDMs previously stimulated with EV_HD_ or EV_HIV_ was collected and added to J-LAT cells (clone 10.6), cells of a Jurkat-derived clone containing latent HIV-1 in single integration site engineered to express green fluorescent protein (GFP) when latency is reverted (59). Since J-LAT cells do not express Gal-1 themselves (Fig. S3), they represent an attractive model to analyze the effect of extracellular Gal-1 stimulation. Fluorescence-activated cell sorting (FACS) analysis of J-LAT cells exposed to conditioned medium from EV_HIV_-stimulated macrophages revealed considerably greater HIV-1 latency reversal than in cells cultured with conditioned medium from macrophages stimulated with EV_HD_ (Fig. 3A and B). Interestingly, 2-fold serial dilutions showed a dose-response effect unveiling two functionally different groups, namely, strong and weak inductors (Fig. S4A and B). Interestingly, direct stimulation of J-LAT with EVs did not trigger HIV latency reversal (Fig. S4C). Since conditioned medium contains not only Gal-1 but also proinflammatory cytokines and other soluble factors that could modulate HIV-1 latency reversal, we evaluated the specific contribution of Gal-1 by silencing Gal-1 expression in MDMs. Silencing efficiency with two different short hairpin RNAs (shRNAs) was determined by qPCR and was revealed to be more than 90% (Fig. 3C). Next, control (scrambled [SCR]) or Gal-1-silenced MDMs were stimulated with EV_HIV_ for 24 h. Conditioned medium was then collected and added to J-LAT cells. We found that silencing Gal-1 in MDMs prior to stimulation with EV_HIV_ significantly reduced reactivation of latent HIV infection (Fig. 3D and E), indicating that Gal-1 secretion by EV-treated macrophages controls HIV latency reversal.

10.1128/mbio.00611-22.3 FIG S3 J-LAT cells do not express Gal-1. Shown are results of immunoblot analysis of Gal-1 expression by J-LAT cells in two independent cell lysates. Results from two independent experiments are shown. Download FIG S3, EPS file, 1.7 MB. Copyright © 2022 Rubione et al. 2022 Rubione et al. https://creativecommons.org/licenses/by/4.0/ This content is distributed under the terms of the Creative Commons Attribution 4.0 International license.

10.1128/mbio.00611-22.4 FIG S4 HIV latency reversal is induced by extracellular vesicles. (A) Latency reversal in J-LAT cells (3 × 10^4^) was determined by analyzing the percentage of eGFP-positive cells by FACS at 24 h after stimulation with concentrated plasma-derived EVs from HIV^+^ donors. PMA (0.5 ng/mL) and PBS were used as positive and negative controls, respectively. (B and C) Conditioned media from MDMs stimulated with EV_HIV_ (four donors, purple, light blue, blue, and pink dots) or EV_HD_ (black dots) were titrated by 2-fold serial dilutions and classified as strong (B) or weak (C) inductors based on eGFP expression by J-LAT cells 24 h poststimulation. *P* values were calculated using one-way ANOVA (A). ****, *P* < 0.0001. Download FIG S4, EPS file, 2.2 MB. Copyright © 2022 Rubione et al. 2022 Rubione et al. https://creativecommons.org/licenses/by/4.0/ This content is distributed under the terms of the Creative Commons Attribution 4.0 International license.

To further dissect the contribution of Gal-1 to the modulation of the inflammatory response triggered by EV_HIV_, we performed a cytokine bead array on conditioned medium from SCR and Gal-1-silenced MDMs before and after EV_HIV_ stimulation. Analysis of basal vehicle-treated cultures revealed no significant changes between the experimental conditions in tumor necrosis factor alpha (TNF-α), IL-6, IL-10, or IL-8, while IL-1β and IL-12p70 showed a slight yet significant increase in Gal-1-silenced MDMs (Fig. S5A). Interestingly, Gal-1-silenced MDMs showed a clear reduction in TNF-α and IL-1β relative to SCR MDMs after EV_HIV_ stimulation (Fig. 3F and G), while other cytokines exhibited no significant changes (Fig. S5B).

10.1128/mbio.00611-22.5 FIG S5 Determination of cytokines in conditioned medium from Gal-1-silenced macrophages before and after stimulation with EVs from HIV^+^ individuals. (A-B) Gal-1-silenced MDMs were stimulated with PBS (A) or EV_HIV_ (B), and TNF-α, IL-1β, IL-6, IL-10, IL-12p70, and IL-8 concentrations were analyzed by a cytokine bead array. Download FIG S5, EPS file, 2.1 MB. Copyright © 2022 Rubione et al. 2022 Rubione et al. https://creativecommons.org/licenses/by/4.0/ This content is distributed under the terms of the Creative Commons Attribution 4.0 International license.

### A glycan-dependent Gal-1-driven pathway favors HIV-1 latency reversal.

**FIG 4:**
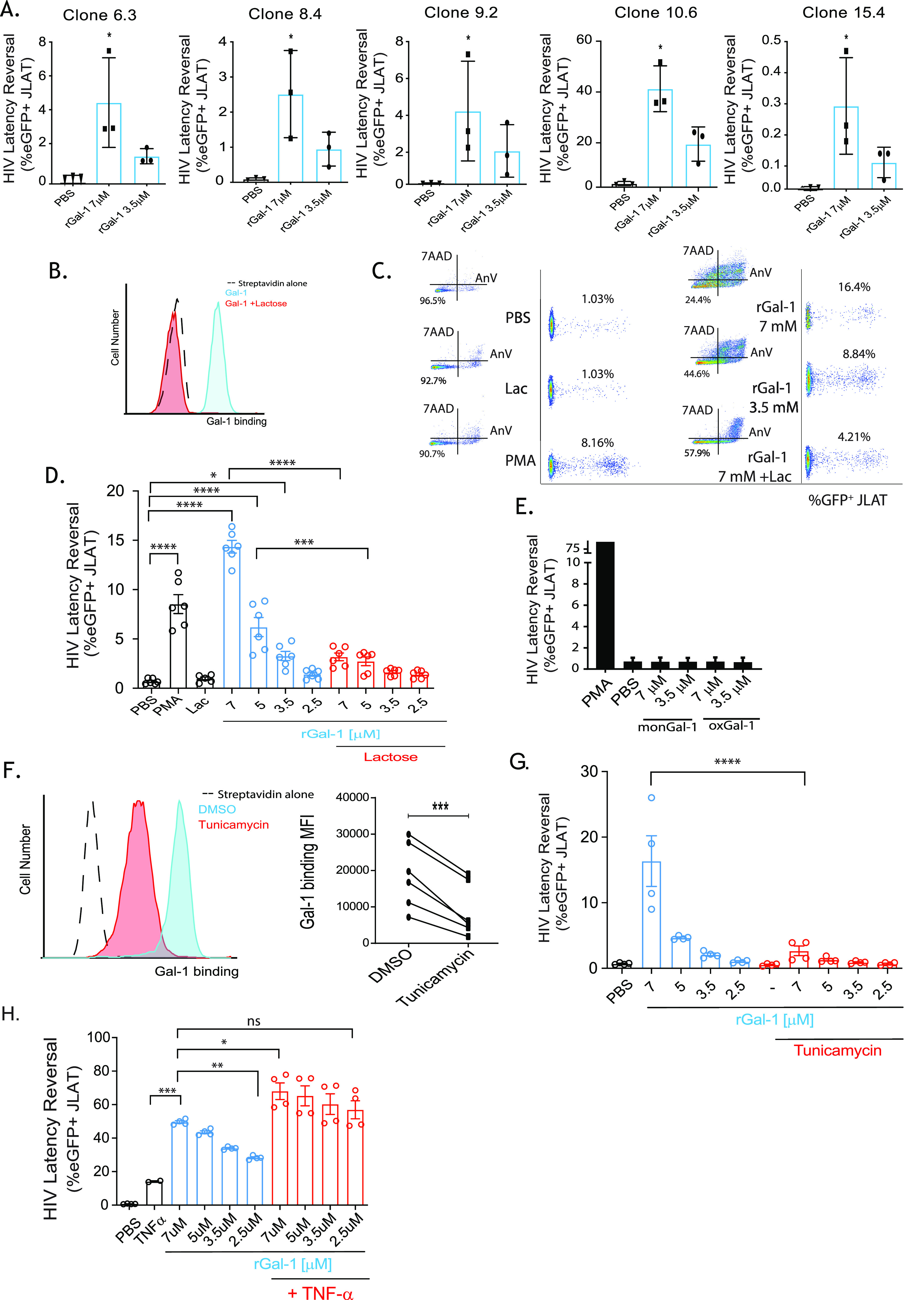
Gal-1 promotes HIV latency reversal in the J-LAT cell model in a glycan-dependent fashion. (A) The effect of rGal-1 on HIV-1 latency reactivation was analyzed in five different clones of J-LAT cells (clones 6.3, 8.4, 9.2, 10.6, and 15.4). (B) Binding of Gal-1 to the surface of J-LAT cells (clone 10.6) was analyzed by incubating 1 × 10^5^ cells with biotinylated Gal-1 (12.5 μg/mL) in the absence or presence of lactose (Lac; 30 mM) for 1 h at 4°C. After extensive washing, cells were incubated with streptavidin-Alexa Fluor 647 (SA) and binding of Gal-1 to the cell surface was analyzed by FACS. (C and D) Latency reactivation in J-LAT cells (clone 10.6; 3 × 10^4^) stimulated with the indicated doses of rGal-1 in the absence or presence of lactose (30 mM) was determined by quantifying the percentage of eGFP-positive cells in the annexin V^−^/7-AAD^−^ gate by FACS at 24 h poststimulation. PMA (0.5 ng/mL) was used as a positive control and PBS as negative control. Representative dot plots (C) and pooled data from 6 experiments (D) are shown. (E) The effects of oxidized Gal-1 (oxGal-1) and monomeric Gal-1 (monGal-1) on HIV-1 latency reactivation were analyzed. PMA (5 ng/mL) was used as a positive control. (F and G) J-LAT cells were treated with the N-glycosylation inhibitor tunicamycin (4 μg/mL) for 18 h at 37°C. Representative histograms showing the binding of recombinant Gal-1 (rGal-1) (left) and the quantification of six independent experiments (right) are shown (F). Cells were incubated with the indicated doses of rGal-1 for 24 h, and latency reversal was analyzed by determining eGFP expression by FACS (from 2 independent experiments) (G). (H) HIV-1 latency reversal in J-LAT cells (3 × 10^5^; clone 10.6) stimulated with TNF-α (0.03 ng/mL), different rGal-1 concentrations, or both are shown (from 2 independent experiments). *P* values were calculated using one-way ANOVA (A, D, G, H) or paired Student’s *t* test (F). ns, nonsignificant; DMSO, dimethyl sulfoxide. *, *P* < 0.05; **, *P* < 0.01; ***, *P* < 0.001; ****, *P* < 0.0001.

To further confirm the role of Gal-1 in HIV latency reversal in J-LAT cells, we next stimulated five different clones of this reporter cell line (6.3, 8.4, 9.2, 15.4, and 10.6, each clone exhibiting a different HIV integration site) with recombinant Gal-1 (rGal-1). We found dose-dependent latency reversal in all clones exposed to rGal-1 (Fig. 4A).

Given the contribution of glycosylated ligands to the extracellular functions of Gal-1 (33, 37), we next assessed the carbohydrate-dependent binding of Gal-1 to J-LAT cells (clone 10.6) using lactose (30 mM), a galectin-specific competitive disaccharide (Fig. 4B). Interestingly, rGal-1 elicited dose-dependent HIV reactivation (Fig. 4C and D), which was prevented by addition of this saccharide. As expected, rGal-1 stimulation also influenced cell viability in a dose-dependent fashion as measured by annexin V and 7-aminoactinomycin D (7-AAD) staining (Fig. 4C and Fig. S6). Since the reduced and dimeric forms of Gal-1 are critical for most extracellular functions of this protein (60), we evaluated an oxidized form of Gal-1 (oxGal-1) (61) and a stable monomeric Gal-1 mutant (monGal-1 [V5D]) as potential regulators of HIV latency reversal. Our results showed that both monGal-1 and oxGal-1 failed to reactivate HIV-1 in J-LAT cells (Fig. 4E), thus emphasizing the importance of the redox status and dimerization balance in Gal-1-driven HIV latency reversal. Finally, pretreatment of J-LAT cells with tunicamycin, an inhibitor of the N-glycosylation pathway, markedly reduced binding of Gal-1 (Fig. 4F) and prevented Gal-1-driven latency reversal (Fig. 4G) in J-LAT cells, highlighting the importance of cell surface N-glycans in Gal-1-mediated reactivation of latent HIV-1 infection.

10.1128/mbio.00611-22.6 FIG S6 Viability of J-LAT cells after rGal-1 treatment. J-LAT cells (clone 10.6; 3 × 10^4^) were stimulated with indicated concentrations of rGal-1 in the absence (dark gray) or presence (light gray) of lactose (30 mM). The percentage of live cells was analyzed in the annexin V^−^/7-AAD^−^ gate by FACS at 24 h poststimulation. *P* values were calculated using one-way ANOVA. ***, *P* < 0.001. Download FIG S6, EPS file, 1.8 MB. Copyright © 2022 Rubione et al. 2022 Rubione et al. https://creativecommons.org/licenses/by/4.0/ This content is distributed under the terms of the Creative Commons Attribution 4.0 International license.

Based on these findings, we evaluated whether TNF-α could potentiate Gal-1-driven HIV latency reversal. As expected, stimulation of J-LAT cells (clone 10.6) with TNF-α (0.03 ng/mL) efficiently reversed HIV latency. Interestingly, simultaneous stimulation with TNF-α and different rGal-1 concentrations not only potentiated the effect of this proinflammatory cytokine but also lowered the threshold of Gal-1 stimulation, as the effect of rGal-1 at its lowest dose (2.5 μM) was similar to that at its highest concentration (7 μM) when used alone (Fig. 4H).

Thus, dimeric Gal-1, released by EV-stimulated macrophages, promotes HIV-1 latency and reversal in a glycan-dependent fashion.

### HIV-1 latency reversal driven by Gal-1 involves activation of the NF-κB pathway.

**FIG 5:**
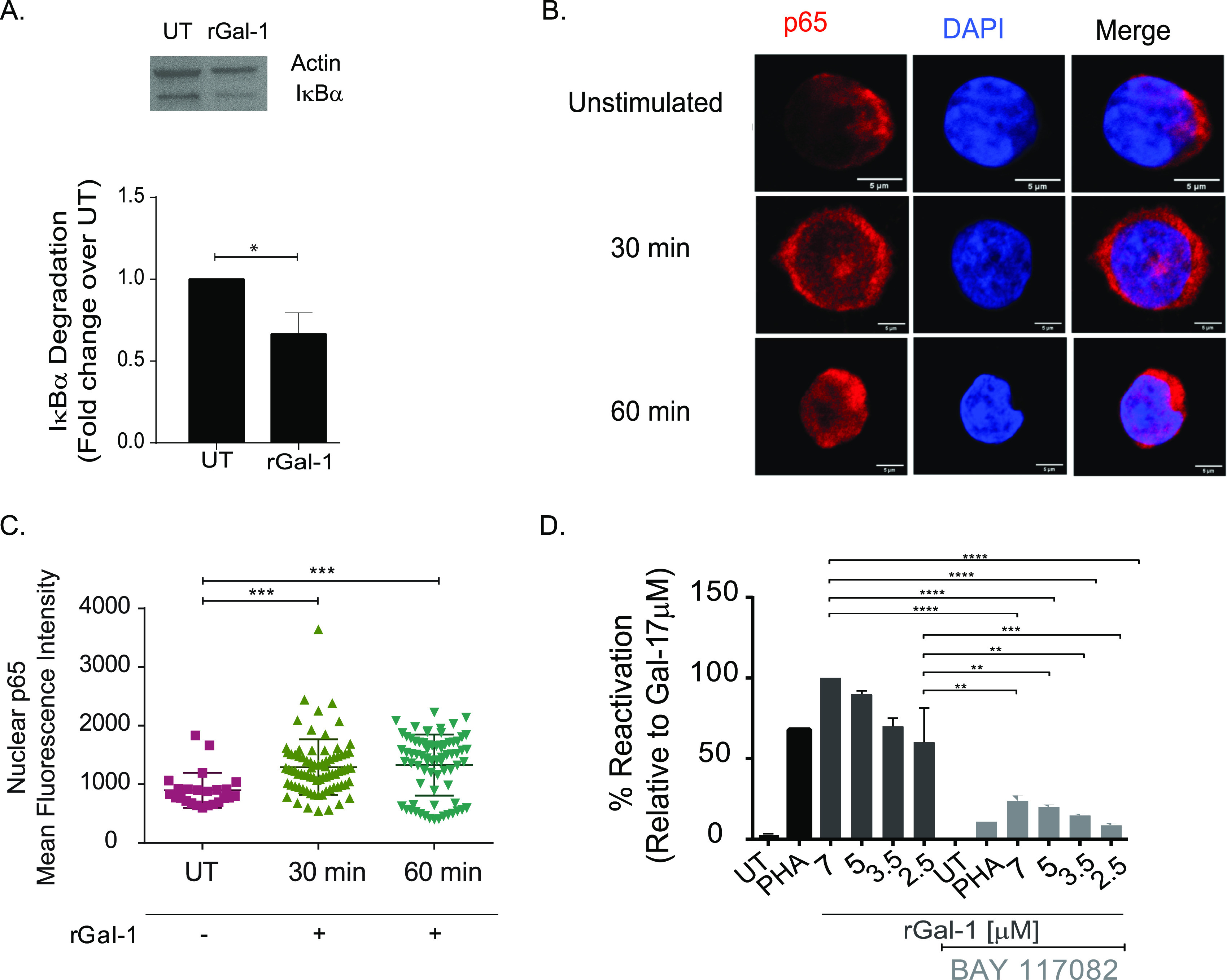
Gal-1 induces HIV latency reactivation through modulation of the NF-κB pathway. (A) Cytosolic IκB-α expression was analyzed by immunoblotting in J-LAT cells stimulated with rGal-1 for 1 h or left unstimulated (UT). A representative blot (top) and the quantification of 3 blots (bottom) are shown. (B and C) Induction of NF-κB activity in J-LAT cells, as determined by quantification of p65 nuclear translocation, analyzed by indirect immunofluorescence. A representative image (B) and the analysis of the mean nuclear fluorescence of ~60 cells per condition performed by blinded operators (C) are shown. (D) J-LAT cells were exposed to an NF-κB inhibitor (BAY-117082; 3 μg/mL) or left untreated for 30 min prior to stimulation with increasing doses of rGal-1. HIV-1 latency reactivation was determined at 24 h poststimulation by determining the percentage of GFP-positive cells by FACS. *P* values were calculated using one-way ANOVA (C and D) or paired Student’s *t* test (A). *, *P* < 0.05; **, *P* < 0.01; ***, *P* < 0.001; ****, *P* < 0.0001.

HIV-1 transcription is dependent on the activation of host transcription factors, with NF-κB playing a prominent role in this process (62, 63). Given the relevance of NF-κB in Gal-1 function (35, 64), we hypothesized that Gal-1 action on HIV-1 latency reversal is mediated by NF-κB activation. To test this hypothesis, we analyzed expression of IκB-α, a regulatory protein that interrupts NF-κB activation by trapping this transcription factor within the cytoplasmic compartment. Exposure to rGal-1 reduced IκB-α expression in J-LAT cells (Fig. 5A), suggesting its ability to trigger NF-κB activation in HIV latently infected cells. This effect was further confirmed by analyzing nuclear translocation of p65 by immunofluorescence (Fig. 5B and C). Furthermore, treatment with an NF-κB inhibitor (BAY 117082) (Fig. 5D, light gray bars) inhibited latency reversal driven both by Gal-1 and l-phytohemagglutinin (PHA-L), a positive control, thus substantiating the relevance of the NF-κB signaling pathway in Gal-1 effects. Thus, Gal-1 promotes glycan-dependent HIV-1 latency reactivation via activation of the NF-κB pathway.

### Gal-1 promotes HIV latency reversal in primary CD4^+^ T cells.

**FIG 6:**
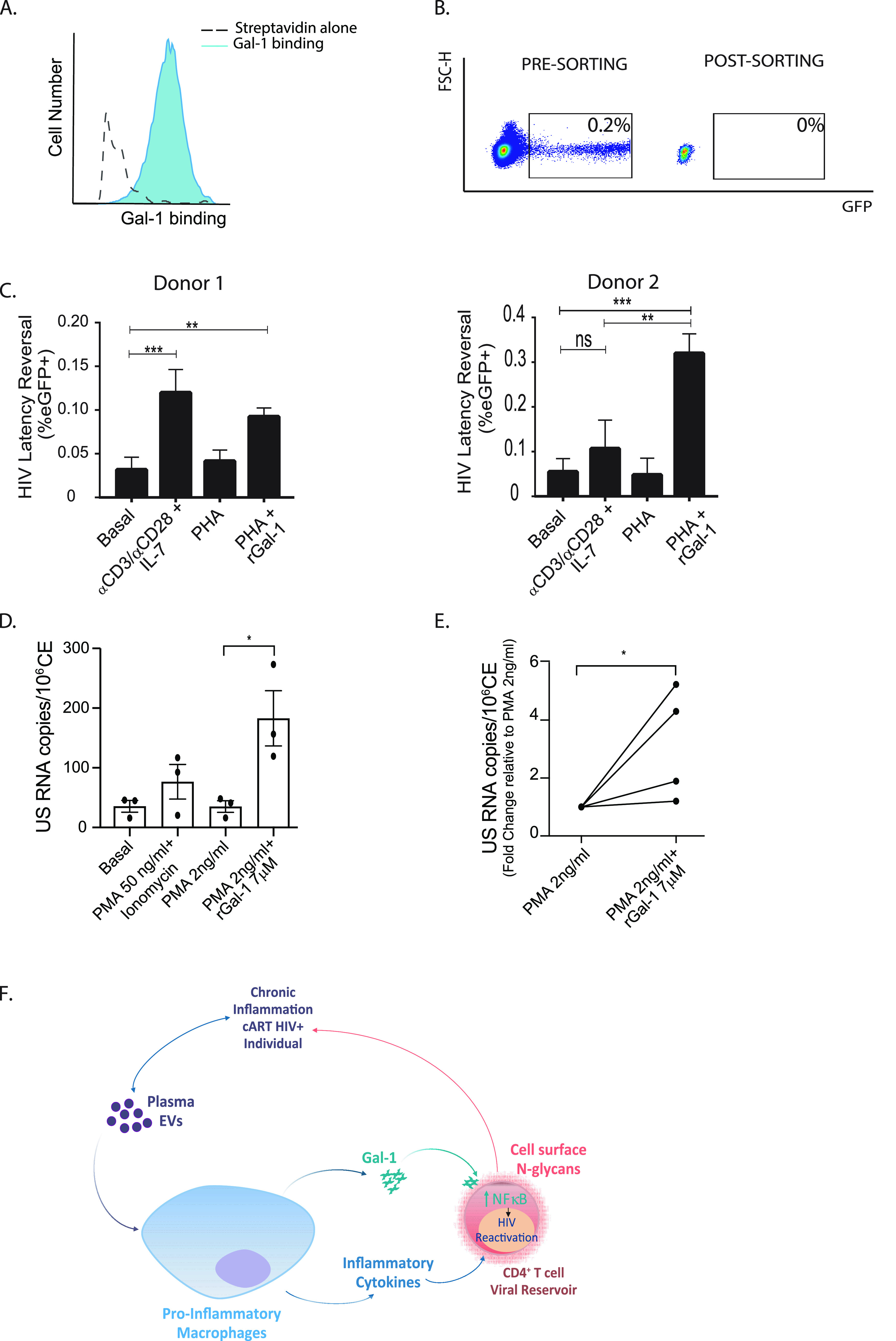
Gal-1 induces HIV-1 latency reversal in human primary CD4^+^ T cells. (A) Gal-1 binding to human CD4^+^ T cells was analyzed by flow cytometry. Streptavidin alone served as a control (SA alone). (B) Resting CD4^+^ T cells were stimulated with CCL19 (100 nM) for 24 h and infected with HIV-EGFP. On day 5 postinfection, GFP^+^ cells were removed by FACS and GFP^−^ CD4^+^ T cells were kept for further experiments. (C) GFP^−^ CD4^+^ T cells were stimulated with either anti-CD3/-CD28 MAb plus IL-7 (50 ng/mL), PHA (1 μg/mL), or PHA (1 μg/mL) plus rGal-1 (7 μM) or left untreated as a control (basal). GFP expression was analyzed at day 3 poststimulation by flow cytometry. (D and E) *Ex vivo* HIV latency reversal was assessed in CD4^+^ T cells from HIV-infected individuals under cART. Cells were purified and cultured for 16 h in the absence of stimuli (basal) or in the presence of PMA (50 ng/mL) and ionomycin (1 μg/mL) as a positive control and stimulated with suboptimal concentrations of PMA (2 ng/mL) either combined or not with rGal-1 (7 μM). A representative graph from 1 patient (D) and pooled data from 4 patients are shown (E). (F) Working model. EVs circulating in the blood of HIV-1-infected individuals stimulate macrophages to secrete both proinflammatory cytokines and Gal-1. Gal-1, in turn, binds to latently infected CD4^+^ T cells in a glycan-dependent fashion and triggers activation of the NF-κB pathway, resulting in HIV latency reversal. Subsequently, low levels of viral replication or the expression of viral pathogen recognition molecular patterns (PAMPs) boosts the inflammatory response. Thus, a regulatory circuit mediated by EVs and Gal-1 controls reservoir dynamics during HIV-1 infection. *P* values were calculated using one-way ANOVA (B and D) or Student’s *t* test (E). *, *P* < 0.05; **, *P* < 0.01; ***, *P* < 0.001.

To study the effects of Gal-1 in reversing HIV-1 latency in a more physiologic setting, we first confirmed binding of this lectin to the surface of human primary CD4^+^ T cells (Fig. 6A) and analyzed its function in an *in vitro* model of HIV latency (65, 66). Briefly, resting CD4^+^ T cells isolated from the blood of healthy donors were pretreated with CCL19, a facilitator of the establishment of latent HIV infection for 24 h. Cells were then infected with a high multiplicity of infection of HIV-1-GFP. At day 5 postinfection, cells were sorted by FACS to remove GFP-positive cells (productively infected) (Fig. 6B). GFP-negative cells (including uninfected and latently infected cells) were exposed to different stimuli to reactivate latent HIV-1. As expected (65, 66), stimulation with anti-CD3/CD28 monoclonal antibody (MAb) and IL-7 (50 ng/mL) reversed HIV latency, as revealed by the increase in GFP expression (Fig. S7). Although purified rGal-1 was not sufficient to trigger HIV latency reversal when added alone to primary cultures, this lectin significantly potentiated reactivation of cells when they were cotreated with suboptimal doses of the activating stimulus PHA (Fig. 6C).

10.1128/mbio.00611-22.7 FIG S7 Flow cytometry of GFP expression after activation of GFP-CD4^+^ T cells. GFP-CD4^+^ T cells were stimulated either with anti-CD3/-CD28 MAb plus IL-7 (50 ng/mL) or with rGal-1 (7 μM) or left untreated as a control (basal). GFP expression was analyzed at day 3 poststimulation by flow cytometry. Download FIG S7, EPS file, 1.3 MB. Copyright © 2022 Rubione et al. 2022 Rubione et al. https://creativecommons.org/licenses/by/4.0/ This content is distributed under the terms of the Creative Commons Attribution 4.0 International license.

To further understand the physiopathological relevance of Gal-1-driven latency reversal, we performed an *ex vivo* assay on CD4^+^ T cells obtained from peripheral blood samples of cART-treated HIV-infected individuals (Fig. 6D and E). Consistent with the results obtained in the *in vitro* model, while suboptimal activation with phorbol myristate acetate (PMA) was not sufficient to induce viral transcription activity, as revealed by US-RNA quantification, costimulation with PMA and rGal-1 resulted in increased US-RNA detection (Fig. 6E). Together, *in vitro* and *ex vivo* experiments on primary CD4^+^ T cells support a role for Gal-1 in HIV-1 latency reactivation.

Collectively, our results indicate that Gal-1 secretion by macrophages triggered by EVs promotes latency reversal of infected CD4^+^ T cells in an N-glycan-dependent fashion (Fig. 6F).

## DISCUSSION

Latent reservoirs are still the main barrier to achieve eradication of HIV. Likewise, chronic immune activation accentuates infection, even during effective cART, promoting serious clinical complications, known as non-AIDS-related comorbidities. Moreover, chronic inflammation drives viral persistence (21). The latent reservoir is comprised by resting CD4^+^ T cells that carry integrated replication-competent provirus, which do not produce significant progeny in cART-treated individuals (2, 21, 67). However, discontinuation of cART results in relapse in viral load from the reservoirs. Understanding the factors that govern the persistence and dynamics of the HIV reservoir constitutes an area of intensive research (68).

Latently infected CD4^+^ T cells can be intermittently activated by antigen recognition and bystander signals derived from inflammatory processes (69). Indeed, HIV persistence, chronic immune activation, and chronic inflammation are interdependent processes that form a vicious cycle in which inflammatory mediators, such as IL-6 and TNF-α, promote latency reversal from latently infected CD4^+^ T cells (6, 70). The subsequent residual levels of viral replication not only stimulate the immune system, promoting inflammation and immune activation, but also contribute to the continuous replenishment of the latent reservoir, even during cART (71).

In this work, we demonstrated that circulating Gal-1 is elevated in HIV-1-infected individuals compared to that in healthy controls. Interestingly, increased serum levels of Gal-1 are independent of immunological, virological, and treatment status. Indeed, we show that increased Gal-1 levels remain intact after treatment initiation, despite the effectiveness of cART to reduce viral load and to restore CD4^+^ T cell counts. These results are in agreement with previous transcriptional studies showing that *LGALS1* mRNA does not change after treatment initiation (52). The persistence of Gal-1 despite cART treatment suggests that the production of this lectin is triggered by early pathogenic changes that occur before treatment initiation which cannot be reverted by decreasing viral replication. The persistence of high levels of circulating Gal-1 during HIV infection parallels that of several inflammatory markers, such as C-reactive protein (CRP) and sCD14 (53), as well as IL-6, granulocyte-macrophage colony-stimulating factor (GM-CSF), CCL11, IL-1β, and IL-8 (54), which are also unaltered or are marginally affected by cART-treatment. Although the mechanisms underlying sustained Gal-1 production in treated individuals remain unknown, this observation is consistent with our previous data showing that EV_HIV_ from cART-treated individuals sustain macrophage production of proinflammatory cytokines (32). Moreover, we found that Gal-1 expression by macrophages hierarchically impacts TNF-α and IL-1β secretion after EV stimulation and that Gal-1 and TNF-α may act together to revert HIV latency. Thus, we hypothesized that circulating EVs might confer on macrophages the ability to secrete Gal-1 as well as proinflammatory cytokines in cART-treated HIV-1-infected individuals. It is noteworthy that increased levels of circulating Gal-1 have been detected in sera from patients suffering other inflammatory conditions, including rheumatoid arthritis (48) and sepsis (44), as well as in samples from experimental autoimmune orchitis (72) and osteoarthritis (73). On the other hand, other studies showed that under some conditions, like celiac disease and allergies, upregulation of Gal-1 acts as a homeostatic mechanism to counterbalance inflammation (40, 74). Future studies should be aimed at examining whether Gal-1 may serve as a compensatory mediator that controls resolution of inflammation during HIV-1 infection, similar to its role in models of neuroinflammation (42) or sialadenitis (75), or whether it may act as an alarmin, thus amplifying inflammatory responses and recapitulating its early role in sepsis (44).

Increased Gal-1 levels in plasma from cART-treated individuals positively correlated with the size of the HIV reservoir, as quantified by viral US-RNA. Our *in vitro* data provide mechanistic insights into this observation, showing that extracellular Gal-1 interacts with latently infected CD4^+^ T cells in a glycan-dependent manner, promoting NF-κB activation and favoring HIV-1 latency reversal. The transcription factor NF-κB is a positive regulator of HIV-1 gene expression, acting in concert with activating protein 1 (AP-1) and nuclear factor of activated T cells (NFAT) to control transcription of the *IL-2* gene (76). These findings suggest that Gal-1-driven NF-κB activation could be the cornerstone of the association between latency reactivation and chronic immune activation and inflammation during HIV infection. Thus, interruption of Gal-1–glycan interactions in HIV-infected individuals would lead to lower NF-κB activation and decreased reversal of the HIV-1 reservoir under inflammatory conditions.

Previous studies showed that Gal-1 promotes the interaction between the viral envelope glycoprotein (gp120) and the cellular receptor CD4, thus facilitating virus attachment and infection (45). Together with the results presented in this study, these findings indicate that Gal-1 may play distinct roles at different stages of HIV infection. Whereas it promotes viral replication by favoring CD4^+^ T cell infection in untreated individuals (45), it could promote latency reactivation in patients under cART treatment. In this sense, we focused on the role of Gal-1 as a mediator of macrophage-driven chronic inflammation. Particularly, we found that macrophages stimulated with EVs from HIV-infected individuals under cART secrete larger amounts of Gal-1, which controls transcriptional activity of J-LAT cells. Notably, previous work has shown that Gal-1 (but not Gal-3) can facilitate HIV-1 infection in monocyte-derived macrophages by sustaining early events of the virus replicative cycle, including adsorption and entry (77), indicating that Gal-1 could influence viral infection by modulating both the T cell and macrophage compartments. Importantly, *in vivo* Gal-1 plasma levels correlated with critical inflammatory markers, including sCD14 and IP-10, and were associated with viral transcription activity, as revealed by US-RNA quantification. Interestingly, Gal-9, a tandem-repeat member of the family, has been associated with HIV transcription *ex vivo* (47) through activation of T cell receptor (TCR)-dependent extracellular signal-regulated kinase 1/2 (ERK1/2) signaling (78), suggesting that Gal-1 and Gal-9, two structurally unrelated members of the galectin family, may act in concert to regulate HIV latency reactivation. While both lectins can interact with latently infected cells, their prominent differences in structure, affinities, and glycan recognition preferences suggest complementary roles of Gal-1 and Gal-9 in HIV-1 latency reactivation. Since several galectins have been identified in many different cells and tissues (79), further studies are warranted to investigate whether a glycosylation-dependent network, composed by anti- or proinflammatory galectins, ultimately governs virus latency and reactivation.

Several viruses, e.g., human T-lymphotropic virus 1, influenza virus type A, herpes simplex virus 1, and Epstein-Barr virus, have been shown to upregulate Gal-1 expression during the course of infection (80–83). In contrast, other viruses, such as dengue virus, reduce Gal-1 expression in different cell types (84). Our results show that HIV infection indirectly induces the secretion of Gal-1 by macrophages, in a process dependent on the stimulation of these cells with circulating EVs. Notably, and even though EVs have been implicated in different aspects of HIV pathogenesis (28), their role in HIV latency and reactivation is controversial. Whereas a series of studies have shown that EVs from either uninfected (85) or HIV-infected (86, 87) cells can reactivate latent HIV infection, another study has recently shown that eliminating EVs from cultures may reverse HIV latency in U1 and ACH2 cell lines (88). Although the mechanisms underlying these discrepancies are still uncertain, differences in EV isolation methods or the presence of viral factors in EV preparations could account for these different effects. While our results suggest that EVs do not directly trigger HIV-1 latency reactivation (Fig. S4C), they show that circulating EVs may trigger Gal-1 secretion by MDMs and that secreted Gal-1 may interact with the viral reservoir in a glycan-dependent manner both in the J-LAT model and in latently infected primary CD4^+^ T cells *in vitro* and *ex vivo* (Fig. 4 and 6C to E). Furthermore, our results show that EV_HIV_ and EV_HD_ have similar size distributions (Fig. 2D and E and Fig. S2) as well as mean plasma concentrations that differed less than 2-fold (Fig. 2F), suggesting that the ability of EV_HIV_ to promote latency reversal (Fig. 3A and B and Fig. S4) and accentuate a proinflammatory milieu (Fig. 2J, Fig. 3F and G, and Fig. S5) was mainly associated with qualitative differences (i.e., cargo and/or surface expression markers) rather than small quantitative variations.

Collectively, our results reveal a coordinated action of EVs and Gal-1 in reprogramming HIV-1 latency reactivation by linking macrophages, inflammation, and infected T cells (Fig. 6F). This study could open a path to tackle both inflammation and viral reservoirs, two major complications in cART-treated HIV-1-infected individuals, using Gal-1-targeted agents, including small-molecule glycan inhibitors, polysaccharide derivatives, peptidomimetics, or Gal-1-specific MAb (79, 89).

## MATERIALS AND METHODS

### Study participants.

A total of 64 subjects participated in this study: 13 healthy HIV-seronegative donors (HD) and 51 HIV-infected individuals, of whom 15 were enrolled during primary HIV infection (baseline), 14 were chronically infected and treatment naive (chronic treatment naive), 22 were chronically infected and under treatment (chronic under treatment), and 9 were elite controllers (EC) (Table S1). Subjects were enrolled under the following inclusion criteria: (i) detection of HIV RNA and (ii) treatment-naive status. The chronic treatment naive cohort was defined as subjects with established HIV infection for over 3 years, detectable viral load (VL; >0.50 HIV RNA copies/mL of plasma) and combined antiretroviral therapy (cART) naive, while subjects chronic under treatment were defined as subjects with established HIV infection, undetectable viral load (<0.50 HIV RNA copies/mL of plasma), and under cART for at least 1 year. EC were defined as subjects infected for more than 5 years with undetectable VL (<50 HIV RNA copies/mL of plasma) and CD4^+^ T cell counts of 0.450 cells/mL of blood and who were cART naive and had no record of opportunistic infections and/or AIDS-related diseases.

To isolate plasma EVs, HIV-positive individuals were recruited at the Fundación Huésped Medical Center (Buenos Aires, Argentina). Blood samples were collected in citrate anticoagulant tubes and processed within 1 h of venipuncture to isolate plasma for EV purification. Healthy donors were voluntary blood donors at Blood Center of the Julio Mendez Hospital (Buenos Aires, Argentina). All healthy donors were individuals older than 18 years who had completed and passed a survey on blood donation and were screened for serological markers before being accepted as donors.

### Antibodies and reagents.

The following antibodies were used: Alexa 594-anti-rabbit, Alexa 647-anti-rabbit, streptavidin Alexa 674 and Alexa 594, and anti-human IgG (Jackson ImmunoResearch Laboratories, Inc.); rabbit polyclonal anti-mouse IΚB-α (Santa Cruz Biotechnology); and mouse anti-CD63 and mouse anti-CD9 (BD Bioscience). The rabbit polyclonal anti-Gal-1 antibody was obtained as previously described (90). Recombinant human Gal-1 (rhGal-1) and its monomeric variant (V5D) were produced and purified as described previously (90, 91). To obtain the oxidized form, rhGal-1 was treated with H_2_O_2_ as previously described (61). In all cases, biotinylation was carried out by incubating 1 mg of rhGal-1 or its variants with 112 nmol of biotin for 30 min at room temperature. Dialysis was performed overnight in 1 mM 2-mercapthoethanol.

### Cell lines, plasmids, and HIV-1 strains.

The human CD4^+^ T cell line J-LAT (clone 10.6) was obtained from the NIH AIDS Reagent Program, Division of AIDS (NIAID, NIH). J-LAT clones 6.3, 8.4, 9.2, and 15.4 were kindly provided by Kenneth W. Witwer. HEK 293T cells were obtained from the ATCC (CRL-11268). J-LAT cells were cultured in RPMI 1640 supplemented with 10% fetal bovine serum (FBS).

NL4-3-IRES-eGFP, encoding full-length HIV-1 in the pBR322 backbone under the control of a viral long terminal repeat promoter, was kindly provided by F. Kirchhoff (Institute of Molecular Virology, Ulm University Medical Center, Ulm, Germany). shRNA-expressing lentiviruses were obtained from Sigma (Mission shRNA).

### Production of lentiviral particles.

Lentiviruses were produced as described elsewhere (92). In brief, 2.5 × 10^3^ HEK 293T cells were seeded on a flat-bottom 96-well plate. Twenty-four hours later, cells were transfected with a mix of 100 ng of pCMV-dR8.2 DVpr, 100 ng of the target’s specific shRNA in the pLKO.1 backbone, and 10 ng of pCMV–VSV-G per well, using the X-tremeGENE HP DNA transfection reagent (Roche), following the manufacturer’s recommendations. Twenty-four hours later, the medium was replaced, and supernatants containing lentiviral particles were collected at 72 h after transfection and precleared by centrifugation.

### HIV latency reversal assay.

J-LAT cells (clones 6.3, 8.4, 9.2, 10.6, and 15.4) were seeded at 3 × 10^5^ cells/mL in 96-well plates and stimulated with different rhGal-1 concentrations or with PMA/PHA as positive controls. PHA and PMA were used at concentrations of 1 μg/mL and 0.5 ng/mL, respectively, unless otherwise specified. After 24 h, cells were harvested and cell viability was assessed by annexin V and 7-AAD staining. Latency reversal was quantified by flow cytometry as the percentage of cells positive for enhanced green fluorescent protein (eGFP) in the annexin V^−^/7-AAD^−^ gate. Alternatively, cells were pretreated with tunicamycin (4 μg/mL) overnight and then incubated as previously described (93).

### Differentiation of monocyte-derived macrophages (MDMs) and purification of CD4^+^ T cells.

Monocytes were isolated from buffy coats of healthy anonymous donors from the Blood Center of Julio Méndez Hospital in Buenos Aires, Argentina, by Ficoll density gradient centrifugation and positive magnetic isolation using anti-CD14-coated beads (Miltenyi). Isolated monocytes were plated for 2 h without serum and subsequently differentiated into macrophages by culturing in complete RPMI 1640 supplemented with 50 ng/mL of GM-CSF for 5 days.

Resting CD4^+^ T cells were negatively purified using immunodensity negative selection cocktail (RosetteSep human T cell enrichment cocktail immunodensity negative-selection cocktail; StemCell) from buffy coats of healthy donors. Cells were maintained in RPMI 1640 supplemented with 10% FBS (Gibco) in the presence of IL-2.

### Gene expression silencing of primary MDMs.

Silencing of gene expression was performed as described (94). Briefly, 1 × 10^6^ MDMs were seeded in the presence of GM-CSF and lentiviral vectors plus simian immunodeficiency virus (SIV)-like particles in the presence of 8 μg/mL of Polybrene. Forty-eight hours later, transduced cells were selected by the addition of 3 μg/mL of puromycin. Before functional studies, SCR and Gal-1-knocked down (Gal-1-KD) MDMs were harvested and reseeded in 96-well plates at 8,000 cells per well. Conditioned medium from SCR and Gal-1KD MDMs stimulated with EV_HIV_ or EV_HD_ for 24 h was used to analyze HIV latency reversal in J-LAT cells. Conditioned medium was titrated by 2-fold dilutions on 30,000 J-LAT cells as described above (Fig. S3A and B). Then a 1:2 dilution was selected for further assays.

### Isolation of EVs from human plasma.

Isolation of EVs from human plasma was performed as described previously (32), following an adaptation of the original protocol (95). Briefly, whole blood drawn by venipuncture was collected in citrate-containing Vacutainer tubes (BD). Platelet-rich plasma (PRP) was obtained by centrifugation (300 × *g*, 10 min). PRP was supplemented with 200 nM Prostaglandin I2 (PGI2) to avoid platelet activation and centrifuged (2,000 × *g*, 15 min) to obtain cell-free plasma. Plasma (2 mL) was loaded onto a homemade size exclusion chromatography (SEC) column (resin, CL-2B; GE Healthcare; support, 12-mL empty cartridges with 20-μm hydrophobic frits; Applied Separations). Twelve fractions (1 mL each) were eluted using 0.9% NaCl–0.38% sodium citrate. Each fraction was analyzed for the presence of the EV markers CD63 and CD9 and soluble IgG by immunoblot analysis. Additionally, equal volumes of extracts were separated by 12% SDS-PAGE under nonreducing conditions and analyzed for CD63, CD81, and IgG, or under reducing conditions for ALIX, HSP70 and APOA1, and blotted onto polyvinylidene fluoride (PVDF) transfer membranes (Thermo Fisher Scientific). Primary antibodies used were as follows: CD63 (BD Pharmingen; catalog number 556019, clone H5C6), CD81 (BD Pharmingen; catalog number 555675, clone JS-81), ALIX CST (catalog number 92880, clone E6P9B), HSP70 (Enzo; catalog ADI-SPA-810, clone C92F3A-5), and APOA1 (CST; catalog number 3350S, clone 5F4). Primary antibody dilutions used for blotting were 1:10,000 for IgG and 1:1,000 for the rest of the antibodies. Blots were revealed using SuperSignal West Pico PLUS chemiluminescent substrate (Thermo Fisher Scientific), and images were acquired with the BioSpectrum-815 imaging system (UVP). Finally, EV-containing fractions were pooled, concentrated by centrifugation, resuspended in 50 μL of phosphate-buffered saline (PBS), and used within the same day for functional studies.

MDMs (250,000 in 300 μL) were cultured in 48-well plates and stimulated for 24 h with 25 μL of EV_HIV/HD_ concentrate. SCR or Gal-1-silenced MDMs were stimulated with 10 μL of EV_HIV/HD_ concentrate per well. To detect albumin as a possible contaminant in EV samples, a sensitive immunoturbidimetric method was performed using a commercial kit for microalbuminuria diagnosis (Wiener; Microalbumin Turbitest AA). Diluted plasma samples (1:500) were used as positive controls.

### Nanoparticle tracking analysis.

Plasma samples were obtained from EDTA-anticoagulated whole-blood venipuncture from 9 healthy and 9 cART-treated HIV^+^ fasted donors. Plasma EVs were isolated by SEC, followed by centrifugation at 30,000 × *g* for 90 min as described above and resuspension in PBS. The size distribution and concentration of EVs were analyzed using NanoSight NS300 equipment (NanoSight, Amesbury, UK). The analysis was performed using the 532-nm laser and 565-nm long-pass filter, with a camera level of 12 and detection threshold of 5. EVs were diluted in PBS before the analysis.

### DLS analysis.

EV size distribution by dynamic light scattering (DLS) was analyzed as described previously (96). Briefly, EVs from healthy and HIV^+^ donors were purified from 2 mL of pooled plasma (*n* = 4 for each condition) by SEC and concentrated by centrifugation. Samples were diluted in PBS to a final volume of 1,000 μL (1:100). The hydrodynamic diameter (D*_h_*) and the size distribution (polydispersity index [PDI]) of the EV samples were assayed by DLS (Zetasizer Nano-ZS; Malvern Instruments, Worcestershire, UK) at a scattering angle of 173°. The Nano-ZS contains a 4-mW He-Ne laser operating at a wavelength of 633 nm, a digital correlator (ZEN3600), and noninvasive back scatter (NIBS) technology. All samples were analyzed at 4°C. The refractive index (RI) was 1.48, and viscosities were between 1.544 and 1.569 cP (4°C). Results are expressed as means ± standard deviations (SD) from at least six runs for each specimen.

### MRPS.

Plasma samples were obtained from EDTA-anticoagulated whole-blood venipuncture from 3 healthy and 3 cART-treated HIV^+^ fasted donors. Plasma EVs were isolated by SEC followed by centrifugation at 30,000 × *g* for 90 min as described above. Purified EVs were resuspended in 50 μL PBS. Microfluidics resistive pulse sensing (MRPS) measurements were conducted using an nCS1 instrument (Spectradyne, Torrance, CA) (97). EVs were diluted 10^5^ to 10^7^ times in 0.1% Tween 20-PBS, and 5 μL was loaded onto polydimethylsiloxane cartridges (diameter range, 65 nm to 400 nm). About 5,000 events were recorded for each sample. All acquired results were analyzed using the nCS1 data analyzer (Spectradyne). For all samples, user-defined filtering was applied by defining two-dimensional (2D) polygonal boundaries based on transition time and diameter to exclude false-positive signals. An additional 80-nm filter was applied in order to compare samples with different background levels.

### Transmission electron microscopy (TEM).

Plasma samples were obtained from EDTA-anticoagulated whole blood from healthy and cART-treated HIV^+^ fasted donors. Plasma EVs were isolated by SEC followed by centrifugation at 30,000 × *g* for 90 min as described above. Purified EVs were resuspended in 50 μL of PBS.

A droplet of each EV suspension fixed in 2% paraformaldehyde was mounted on a collodion-coated copper grid (400 mesh) for 20 min. Then grids were washed three times with PBS (pH 7.4) and once with distilled water. Samples were incubated with 4% uranyl acetate for 40 s. Finally, grids were visualized under a JEM 1200 EX II transmission electron microscope (JEOL Ltd., Tokyo, Japan) and photographed by an Erlangshen ES1000W camera (model 785; Gatan Inc., Pleasanton, CA) at the Electron Microscopy Central Service of the School of Veterinary Sciences, University of La Plata. Micrographs were analyzed with the ImageJ software for EV size determination.

### HIV-1 load in plasma EVs.

Plasma EVs were obtained as described above and resuspended in 1 mL of PBS. HIV-1 RNA was quantified by Cobas HIV-1 test (Roche Diagnostics) in a Cobas 4800 system, which is a fully automatized system for extraction, amplification, detection, and quantification of nucleic acids by automated real-time HIV-1 assay (Abbott).

### Analysis of rhGal-1 binding and functionality.

To analyze cell surface binding of Gal-1, a total of 200,000 cells were incubated with 1.6 mg of biotinylated rhGal-1 for 60 min at 4°C. Cells were subsequently incubated with conjugated streptavidin for 45 min at 4°C. When indicated, binding of Gal-1 to the cell surface was competed by adding 30 mM lactose to the culture medium. To analyze the functionality of this lectin, cells were incubated with increasing concentrations of rhGal-1 (2.5, 3, 5, and 7 μM) in the absence or presence of lactose (30 mM). Gal-1 concentrations were chosen based on those reaching different dimerization ratios, abundance in different pathophysiologic settings, and previously published studies (33, 35, 36, 38, 60, 61, 91).

### Primary CD4^+^ T cell latency model.

The HIV latency model was established using primary CD4^+^ T cells from healthy donors as previously described (65, 66). Briefly, resting CD4^+^ T cells were cultured for 24 h in RF10 with 100 nM CCL19. Cells were incubated with NL4-3-IRES-eGFP for 2 h at 37°C, washed, and cultured for 5 days in RF10 plus 2 U/mL of IL-2. At day 5 postinfection, T cells were analyzed for eGFP expression (productive infection) and eGFP^−^ T cells were sorted (FACSAria; BD Biosciences). Aliquots of 1 × 10^5^ sorted cells were cultured for an additional period of 3 days (day 8) in 96-well plates in 200 μL of RF10 plus 2 U/mL of IL-2 either alone (spontaneous eGFP expression) or stimulated with anti-CD3/CD2/CD28 MAb-coated beads (Miltenyi). IL-7 (50 ng/mL) was also added to culture media as a latency reactivation positive control. Additionally, cells were cultured in the presence of PHA-L (1 μg/mL) and rGal-1 (7 μM), either alone or in combination. Cells were analyzed for eGFP expression by flow cytometry (FACSCanto; BD Biosciences) at day 8.

### HIV-1 reservoir quantification.

Quantitative real-time PCR for cell-associated (CA) HIV RNA and DNA was performed as follows. CA HIV DNA and unspliced RNA (US-RNA) were determined by real-time PCR on samples obtained once study subjects were on cART. CD4^+^ T cells were isolated from frozen PBMCs using an immunomagnetic selection kit (StemCell Technologies, Canada), and only samples with 90% purity (determined by flow cytometry) were assayed. DNA and RNA were extracted using a Qiagen minikit (AllPrep DNA/RNA minikit; Qiagen, Germany), quantified, and stored at −80°C until use. All samples from each participant were extracted at the same time, run on the same PCR plate, and subsequently analyzed together. Total HIV DNA was determined as described previously (94). Briefly, a single-step real-time PCR was performed using 5 μL of extracted DNA as input per 50 μL of reaction mixture, in triplicate. HIV DNA copy numbers were standardized to cellular equivalents using a CCR5 SYBR green real-time PCR. The lower limit of detection (LLOD) was 1 copy per well. For determination of US-RNA, Pasternak’s protocol was used (58). Briefly, a heminested PCR was performed with 16 cycles of amplification followed by a second amplification round of quantitative real-time PCR. US-RNA copy numbers were standardized to cellular equivalents using an 18S RNA real-time PCR (Invitrogen). The LLOD was 1 copy per well. Each sample was assayed in quadruplicate, and a non-reverse transcriptase control was used to detect DNA contamination. Quantitative real-time PCR assays were run for 40 cycles.

### *Ex vivo* HIV latency reversal.

The HIV latency reversal assay was performed as described previously (47), with minor changes. Briefly, CD4^+^ T cells were isolated from peripheral blood samples (20 mL) from HIV-infected individuals under cART using immunodensity negative-selection cocktail (RosetteSep; StemCell). Cells were plated at 10^6^/mL and cultured for 16 h as follows: no stimulus (basal), PMA (50 ng/mL) plus ionomycin (1 μg/mL) as a positive control, suboptimal PMA (2 ng/mL), and suboptimal PMA (2 ng/mL) plus rGal-1 (7 μM). RNA was extracted using a PureLink RNA minikit (Invitrogen), and US-RNA was quantified as described above.

### qPCR.

Cellular RNAs were isolated using the RNeasy Plus minikit (Qiagen), and 1 μg of RNA was reverse transcribed with Moloney murine leukemia virus (MMLV) reverse transcription (Invitrogen). Only 1:10 cDNA was used for each PCR, performed with SYBR green (Applied Biosystems) on a real-time thermal cycler (Step One Plus; Applied Biosystems). Cycle thresholds (*C_T_*s) were normalized to the *C_T_* of β-actin.

Primer sequences were as follows: β-actin Fw, 5′-AGGCATCCTCACCCTGAAGT-3′; β-actin Rev, 5′-GCGTACAGGGATAGCACAGC-3′; *LGALS-1* Fw, 5′-TCGCCAGCAACCTGAATCTC-3′; and *LGALS-1* Rw, 5′-GCACGAAGCTCTTAGCGTCA-3′.

### Gal-1 and cytokine determinations in serum samples and cell supernatants.

Cytokines (IL-6 and IP-10) were detected by enzyme-linked immunosorbent assay (ELISA) in cell culture supernatants and serum samples, according to the manufacturer’s instructions (BD Biosciences). sCD14 and sCD163 in serum samples were assessed by ELISA according to the manufacturer’s instructions (Thermo Scientific).

A cytokine bead array (CBA; BD Biosciences) was used to determine TNF-α, IL-1β, IL-6, IL-10, IL-12p70, and IL-8 concentrations in SCR and Gal-1-KD MDM conditioned media, according to the manufacturer’s instructions.

Serum Gal-1 was determined using an in-house ELISA as described previously (35). In brief, high-binding 96-well microplates (Costar; Corning) were coated with capture antibody (2 μg/mL of purified rabbit anti-Gal-1 polyclonal IgG) in 0.1 M sodium carbonate (pH 9.5). After incubation for 18 h at 4°C, wells were rinsed three times with washing buffer (0.05% Tween 20 in PBS) and incubated for 1 h at room temperature with blocking solution (2% bovine serum albumin (BSA) in PBS). One-hundred-microliter volumes of samples and standards were diluted in 1% BSA and incubated for 18 h at 4°C. Plates were then washed and incubated with 100 ng/mL of biotinylated detection antibody (purified rabbit anti-Gal-1 polyclonal IgG) for 1 h. Plates were rinsed three times before addition of 0.3 μg/mL horseradish peroxidase (HRP)-labeled streptavidin (Sigma-Aldrich) for 30 min. After a washing, 100 μL of TMB solution (0.1 mg/mL of tetramethylbenzidine and 0.06% H_2_O_2_ in citrate phosphate buffer [pH 5.0]) was added to the plates. The reaction was stopped by adding 4 N H_2_SO_4_. Optical densities were determined at 450 nm in a Multiskan MS microplate reader (Thermo Fisher Scientific). A standard curve ranging from 2.5 to 160 ng/mL of rGal-1 was run in parallel.

### Flow cytometry.

For surface staining, cells were washed once in PBS, followed by incubation for 30 min at 4°C with the corresponding antibody. Prior to FACS analysis, cells were washed twice with PBS. For intracytoplasmic staining, cells were fixed with 4% paraformaldehyde (PFA), washed, and permeabilized according to the manufacturer’s instructions (BD CytoFix and CytoPerm kit). Cells were subsequently incubated with the conjugated antibody for 45 min at room temperature. Cells were acquired on a FACSCanto instrument (BD) and analyzed using FACSDiva software (BD).

### Confocal microscopy.

A total of 10^5^ cells were seeded on poly-l-lysine-coated glass coverslips for 30 min, fixed in 4% paraformaldehyde, quenched with 0.1 M glycine, permeabilized in ice-cooled methanol for 7 min, and incubated with a primary mouse anti-human p65 NF-κB antibody (BD Biosciences) overnight. After extensive washing, cells were incubated with Alexa Fluor 594-labeled donkey anti-mouse secondary antibodies (Jackson ImmunoResearch Laboratories, Inc.). The coverslips mounted with 4′,6-diamidino-2-phenylindole (DAPI) Fluoromount-G (SouthernBiotech) were examined under a Zeiss LSM800 confocal microscope using a Plan Apochromat 60 × 1.42 numerical-aperture (NA) oil immersion objective. Images were analyzed using NIS-Element software.

### Immunoblotting.

Cells were lysed in precooled radioimmunoprecipitation assay buffer (1% Triton X-100, 0.1% SDS, 50 mM Tris [pH 7.5], 150 mM NaCl, and 0.5% sodium deoxycholate) supplemented with a cocktail of protease inhibitors (Roche) and were cleared from nuclei by centrifugation at 15,000 × *g* for 5 min. Equal amounts of protein extracts were separated by 4 to 12% SDS-PAGE and blotted onto a PVDF transfer membrane (Thermo Fisher Scientific) under reducing conditions. Blots were revealed using SuperSignal West Pico chemiluminescent substrate (Thermo Fisher Scientific).

### Bioinformatics analysis and meta-analysis selection criteria.

Studies were selected to analyze and compare *LGALS1* transcript levels in uninfected healthy donors and HIV-infected patients. Studies involving coinfection and/or comorbidity cohorts, analysis of clinical outcomes based on drug interventions, and SIV infection were excluded. All studies included an uninfected control sample. A total of 14 studies including 15 data sets that qualified for our meta-analysis were analyzed. Detailed information can be found in Table S3. Data were analyzed with the GXB software available at http://hiv.gxbsidra.org/dm3/geneBrowser/list.

### Statistical analyses.

Data were analyzed using Prism (GraphPad Software). Normality of the data was tested using the Kolmogorov-Smirnov test. Based on the normality test, either one-way analysis of variance (ANOVA) followed by the Tukey’s honestly significant difference (HSD) posttest or the Kruskal-Wallis test followed by Dunn’s posttest was used for multiple-comparison analyses. Due to the disparity in the FACS values obtained in different experiments, data were normalized to control conditions to show pooled results from several experiments when indicated.

### Ethics statement.

All samples from HIV-infected subjects were collected after written informed consent was obtained. For *in vitro* experiments, CD4^+^ primary T cells were isolated from buffy coats of healthy anonymous donors from the Blood Center of the Julio Méndez Hospital in Buenos Aires, Argentina. All donors were >18 years. In this case, consent was not obtained [exemption 45 CFR 46.101(b), HHS] because samples had not been collected specifically for this research study and were supplied without personal identifiable information. None of the investigators on this research project have any ready means to link the materials back to living individuals.
