# Supplementary material for: A Dynamic Interplay of Circulating Extracellular Vesicles and Galectin-1 Reprograms Viral Latency during HIV-1 Infection
Source: mBio. 2022 Aug 9;13(4):e00611-22. doi: 10.1128/mbio.00611-22 (PMC9426495; doi:10.1128/mbio.00611-22)
Supplement: TABLE S1 [file mbio.00611-22-s0008.pdf]

Supplementary Table 1: Clinical features of patients included in this study.

Condition	Patient ID[a]	Gal-1 (ng/ml)[b]	Viral Load RNA copies/ml	CD4+ T cell count Cells/ml	Gender	Age (years)	AIDS-Relatated Diseases
Healthy Donor	HD1	13.5	NA	NA	NA	>18	NA
	HD2	16.5	NA	NA	F	>18	NA
	HD3	12.5	NA	NA	F	>18	NA
	HD4	11.6	NA	NA	F	>18	NA
	HD5	13.7	NA	NA	F	>18	NA
	HD6	51.4	NA	NA	NA	>18	NA
	HD7	17.6	NA	NA	F	>18	NA
	HD8	79	NA	NA	NA	>18	NA
	HD9	48.6	NA	NA	NA	>18	NA
	HD10	56.8	NA	NA	NA	>18	NA
	HD11	86.5	NA	NA	NA	>18	NA
	HD12	70.8	NA	NA	NA	>18	NA
	HD13	91	NA	NA	NA	>18	NA
Baseline	B1	163.4	180	552	M	27	NO
	B2	143.3	159858	117	F	50	NO
	B3	136.3	19434	327	F	30	NO
	B4	105.8	7643	328	M	31	NO
	B5	302.3	66650	452	F	36	NO
	B6	149	463	881	M	24	NO
	B7	89.8	20106	787	M	58	NO
	B8	148.6	26547	400	M	36	NO
	B9	102.3	406352	347	M	27	NO
	B10	146.8	36338	768	M	48	NO
	B11	223.5	283441	187	M	52	NO
	B12	440.6	>500000	627	M	45	NO
	B13	172.3	>500000	421	M	76	NO
	B14	156.9	>500000	339	M	32	NO
	B15	183.3	>500000	213	M	48	NO
Chronic Treatment Naive	ChTN1	174.6	>500000	13	M	34	NO
	ChTN2	104.5	34395	4	M	32	NO
	ChTN3	184.1	85947	25	F	27	NO
	ChTN4	107.9	101669	NA	M	37	NO
	ChTN5	254.7	179591	16	M	54	NO
	ChTN6	214.3	6723	207	M	54	NO
	ChTN7	124.8	11026	585	M	53	NO
	ChTN8	230.8	169742	717	M	43	NO
	ChTN9	189.1	30830	268	M	27	NO
	ChTN10	143.1	139439	348	M	38	NO
	ChTN11	175	>500000	44	M	54	NO
	ChTN12	91.6	22	828	M	44	NO
	ChTN13	159.1	40	351	M	39	NO
	ChTN14	76.7	NA	NA	M	NA	NO
Chronic Cart	ChcART1	84.3	<50	NA	NA	NA	NA
	ChcART2	54.9	<50	NA	NA	NA	NA
	ChcART3	67.4	<50	NA	NA	NA	NA
	ChcART4	252.6	<50	716	M	46	TBC[c]
	ChcART5	220.6	<50	909	M	53	NO
	ChcART6	116.1	<50	576	F	30	NO
	ChcART7	169.7	<50	979	M	31	NO
	ChcART8	189	<50	1036	M	48	NO
	ChcART9	131.5	<50	702	M	31	NO
	ChcART10	265.3	<50	829	M	58	NO
	ChcART11	180.8	<50	746	M	36	NO
	ChcART12	163.4	<50	523	M	52	NO
	ChcART13	307.2	<50	594	M	45	NO
	ChcART14	300.9	<50	854	M	76	NO
	ChcART15	127.2	<50	691	M	32	NO
	ChcART16	140.7	<50	386	M	48	NO
	ChcART17	137.3	<50	472	F	43	NO
	ChcART18	192	<50	479	M	45	NO
	ChcART19	114.5	<50	635	M	49	NO
	ChcART20	91.4	<50	762	M	33	NO
	ChcART21	146.1	<50	703	M	33	NO
	ChcART22	216.9	<50	6	M	42	NO
ELITE controllers	EC1	263.9	<50	1038	F	42	NO
	EC2	151.6	<40	860	F	44	NO
	EC3	176.5	<40	995	F	43	NO
	EC4	173.8	<50	406	F	40	NO
	EC5	201.3	<50	754	M	29	NO
	EC6	154.7	<50	612	F	50	NO
	EC7	201.3	<50	879	F	54	NO
	EC8	158.5	<40	960	F	42	NO
	EC9	192.5	<50	456	M	30	NO

a HD, Healthy Donor. B,Baseline.ChTN, Chronic Treatment Naïve. ChCART, Chronic under Cart. EC, Elite Controller. b Determined by ELISA. C Diagnosed prior to HIV infection. F, Female. M, Masculine. NA, not available.
