# Supplementary material for: A Dynamic Interplay of Circulating Extracellular Vesicles and Galectin-1 Reprograms Viral Latency during HIV-1 Infection
Source: mBio. 2022 Aug 9;13(4):e00611-22. doi: 10.1128/mbio.00611-22 (PMC9426495; doi:10.1128/mbio.00611-22)
Supplement: TABLE S2 [file mbio.00611-22-s0009.pdf]

Supplementary Table 2: Studies included in LGALS1 mRNA meta-analysis

Study Number	Sample Set	Accession Number	Sample Count
1	Inflammation and macrophage activation in adipose tissue of HIV-infected patients under antiretroviral treatment	GSE19811	13
2	The National NeuroAIDS Tissue Consortium Brain Gene Array: Two types of HIV-associated neurocognitive impairment	GSE35864	72
3	Genome wide mRNA expression correlates of viral control in CD4+T cells from HIV-1 infected individuals	GSE18233	202
4	Chronic CD4+ T cell Activation & Depletion in HIV-1 Infection: Type I Interferon-Mediated Disruption of T Cell Dynamic	GSE9927	20
5	Comparison of transcriptional profiles of CD4+ and CD8+ T cells from HIV-infected patients and uninfected control group	GSE6740	40
6	Expression data from HAART interruption in HIV patients	GSE28177	6
7	Microarray Analysis of Lymphatic Tissue Reveals Stage-Specific, Gene-Expression Signatures in HIV-1 Infection	GSE1636	52
8	Expression data from CD11c+ mDCs in HIV infection	GSE42058	8
9	HIV-1 infection in human PBMCs in vivo	GSE2171	87
10	Transcriptional profiling of CD4 T-cells in HIV-1 infected patients	GSE23879	40
11	Differential Gene Expression in HIV-Infected Individuals Following ART	GSE44228	72
12	Gene-expression profiling of HIV-1 infection and perinatal transmission in Botswana	GSE4124	45
13	Whole Blood Transcriptional Response to Early Acute HIV	GSE29429-GPL6947	185
14	Expression data from HIV exposed and uninfected women	GSE33580	86
14 bis	Whole Blood Transcriptional Response to Early Acute HIV	GSE29429-GPL10558	47
