# Supplementary material for: A Dynamic Interplay of Circulating Extracellular Vesicles and Galectin-1 Reprograms Viral Latency during HIV-1 Infection
Source: mBio. 2022 Aug 9;13(4):e00611-22. doi: 10.1128/mbio.00611-22 (PMC9426495; doi:10.1128/mbio.00611-22)
Supplement: TABLE S3 [file mbio.00611-22-s0010.pdf]

Supplementary Table 3: Clinical and virological data of patients.

Patient ID	Treatment Time (days)	Average tDNA copy number/10^6 CE	Average US-RNA copy number/10^6 CE	Viral Load (copies/ml)	CD4+ T cells Count (cells/ml)	Serum Gal-1 [ng/ml]
P1a	120d	82.95	0.05	2054	432	77.64
P1b	210d	64.42	0.06	50	542	76.11
P1c	300d	174.83	6.98	50	546	74.23
P1d	540d	50.5	92.04	50	973	63.30
P1e	420d	65.35	15	50	700	187.32
P2	60d	254.15	234.1	285	467	154.83
P3a	450d	46.81	79.7	50	1342	86.90
P3b	120d	162.36	11.11	50	978	104.73
P3c	240d	106.12	26.07	50	849	129.98
P4	210d	155.85	2.89	50	371	398.52
P5a	390d	49.58	23.95	50	522	454.23
P5b	210d	153.79	22.04	50	523	141.88
P6a	570d	25.13	2.34	40	612	100.49
P6b	90d	33.56	7.79	50	940	103.36
P7	90d	1040.74	71.86	50	271	153.42
P8	90d	51.91	47.21	50	474	132.97
P9a	210d	295.55	41.03	50	578	125.81
P9b	30d	838.17	12.58	810	367	139.08
P10a	1050d	151.9	21.42	50	532	183.96
P10b	1170d	151.04	54.32	40	472	137.09
P10c	570d	140.54	9.41	50	278	99.96
P10d	690d	192.65	36.85	50	524	136.15
P10e	960d	106.55	21.99	50	562	125.57
P11a	240d	483.2	33.82	50	443	203.82
P11b	360d	557.3	23.48	50	479	191.72
P12a	450d	198.33	12.42	50	635	114.29
P12b	150d	568.48	1.18	50	516	178.09
P12c	240d	204.81	3.53	50	560	116.52
P13a	450d	388.07	5.35	50	762	91.26
P14b	600d	155.06	32.31	66	691	137.55
P15	150d	94.77	77.91	50	535	118.99
P16	240d	394.02	16.44	45	991	135.87
P17	600d	314.29	37.24	40	703	145.90
P18a	390d	65.11	26.57	50	481	286.18
P18b	600d	74.52	262.25	50	1101	281.01
P19	750d	97.54	49.3	40	6	216.51
